# Characterization of cardiac metabolism in iPSC-derived cardiomyocytes: lessons from maturation and disease modeling

**DOI:** 10.1186/s13287-022-03021-9

**Published:** 2022-07-23

**Authors:** Sofija Vučković, Rafeeh Dinani, Edgar E. Nollet, Diederik W. D. Kuster, Jan Willem Buikema, Riekelt H. Houtkooper, Miranda Nabben, Jolanda van der Velden, Birgit Goversen

**Affiliations:** 1 Department of Physiology, Amsterdam University Medical Centers, Amsterdam Cardiovascular Sciences, Location VU Medical Center, 1081 HZ Amsterdam, The Netherlands; 2 Utrecht Regenerative Medicine Center, Circulatory Health Laboratory, Department of Cardiology, University Medical Center Utrecht, University Utrecht, 3508 GA Utrecht, The Netherlands; 3 Laboratory Genetic Metabolic Diseases, Amsterdam University Medical Centers, Amsterdam Gastroenterology, Endocrinology, and Metabolism, Amsterdam Cardiovascular Sciences, University of Amsterdam, 1105 AZ Amsterdam, The Netherlands; 4 Departments of Genetics & Cell Biology and Clinical Genetics, CARIM School for Cardiovascular Diseases, Faculty of Health, Medicine and Life Sciences, Maastricht University Medical Center+, 6200 MD Maastricht, The Netherlands

**Keywords:** iPSC, Stem cells, Cardiomyocyte, Metabolism, Maturation, HCM, Disease modeling

## Abstract

**Background:**

Induced pluripotent stem cell-derived cardiomyocytes (iPSC-CMs) have emerged as a powerful tool for disease modeling, though their immature nature currently limits translation into clinical practice. Maturation strategies increasingly pay attention to cardiac metabolism because of its pivotal role in cardiomyocyte development and function. Moreover, aberrances in cardiac metabolism are central to the pathogenesis of cardiac disease. Thus, proper modeling of human cardiac disease warrants careful characterization of the metabolic properties of iPSC-CMs.

**Methods:**

Here, we examined the effect of maturation protocols on healthy iPSC-CMs applied in 23 studies and compared fold changes in functional metabolic characteristics to assess the level of maturation. In addition, pathological metabolic remodeling was assessed in 13 iPSC-CM studies that focus on hypertrophic cardiomyopathy (HCM), which is characterized by abnormalities in metabolism.

**Results:**

Matured iPSC-CMs were characterized by mitochondrial maturation, increased oxidative capacity and enhanced fatty acid use for energy production. HCM iPSC-CMs presented varying degrees of metabolic remodeling ranging from compensatory to energy depletion stages, likely due to the different types of mutations and clinical phenotypes modeled. HCM further displayed early onset hypertrophy, independent of the type of mutation or disease stage.

**Conclusions:**

Maturation strategies improve the metabolic characteristics of iPSC-CMs, but not to the level of the adult heart. Therefore, a combination of maturation strategies might prove to be more effective. Due to early onset hypertrophy, HCM iPSC-CMs may be less suitable to detect early disease modifiers in HCM and might prove more useful to examine the effects of gene editing and new drugs in advanced disease stages. With this review, we provide an overview of the assays used for characterization of cardiac metabolism in iPSC-CMs and advise on which metabolic assays to include in future maturation and disease modeling studies.

**Supplementary Information:**

The online version contains supplementary material available at 10.1186/s13287-022-03021-9.

## Introduction

Modeling of cardiac disease using stem cell-derived models has advanced during past years and has great potential to define disease mechanisms and to test the toxicity and effectiveness of compounds. A limitation of current induced pluripotent stem cell-derived cardiomyocytes (iPSC-CMs) is their immature, fetal-like nature [28, 45]. In order to increase the resemblance to adult cardiomyocytes, several methods have been developed to mature the morphology and function of iPSC-CMs [1]. During cardiomyocyte development and maturation, cellular metabolism and alterations therein play an important role. Accordingly, metabolism has been shown to be central in maturing iPSC-derived cardiac tissue models [3, 79, 86]. Moreover, perturbations in cellular metabolism are central in the pathogenesis of cardiac disease [51]. Thus, metabolic characterization is key to establish if iPSC-CMs show disease-specific changes as observed in pathological conditions.

Hypertrophic cardiomyopathy (HCM) is an example of a genetic disorder that is characterized by altered metabolism. The disease may be caused by pathogenic gene variants (i.e., mutations) in sarcomere proteins [54] and enzymes involved in cellular metabolism [85]. Sarcomere mutations alter myofilament function [55, 84, 90], which is thought to underlie changes in the energetic status of the heart that has been proposed as an early pathomechanism in HCM. Nuclear magnetic spectroscopy in HCM patients showed decreased cardiac energetics in sarcomere mutation carriers even before the onset of hypertrophy [16]. In addition, increased oxygen consumption has been reported in preclinical sarcomere mutation carriers before the development of hypertrophy, followed by a decrease in oxygen consumption in the hearts of patients with advanced HCM [29, 63]. Changes in energetic status and metabolism are thus present at early and advanced HCM disease stages in sarcomere mutation carriers and are considered as a possible therapeutic target [73, 88].

In disorders caused by mutations in metabolic enzymes and mitochondrial components with an HCM phenotype, the primary defect is impaired cellular metabolism that subsequently causes cardiac hypertrophy [85]. Examples of a primary metabolic-induced HCM phenotype are lysosomal storage diseases (e.g., Anderson–Fabry disease) or primary mitochondrial diseases (e.g., Barth syndrome). In pediatric HCM, > 25% of patients suffer from an inborn error of metabolism, underlining the central role for metabolism in the development of HCM [15].

While altered metabolism represents a central pathomechanism in HCM, relatively limited information is available on metabolic characteristics in HCM iPSC-CMs. Based on a PubMed (MEDLINE) database search strategy, we here provide an overview of maturation studies that quantified certain aspects of iPSC-CM metabolism and describe the metabolic phenotype of reported iPSC-CM HCM models thus far. In addition, based on current knowledge of HCM pathology, we make several suggestions for future research to optimally use iPSC-CMs to decipher metabolic aberrations caused by mutations.

### Excitation–contraction coupling and metabolism in the adult heart

**Fig. 1:**
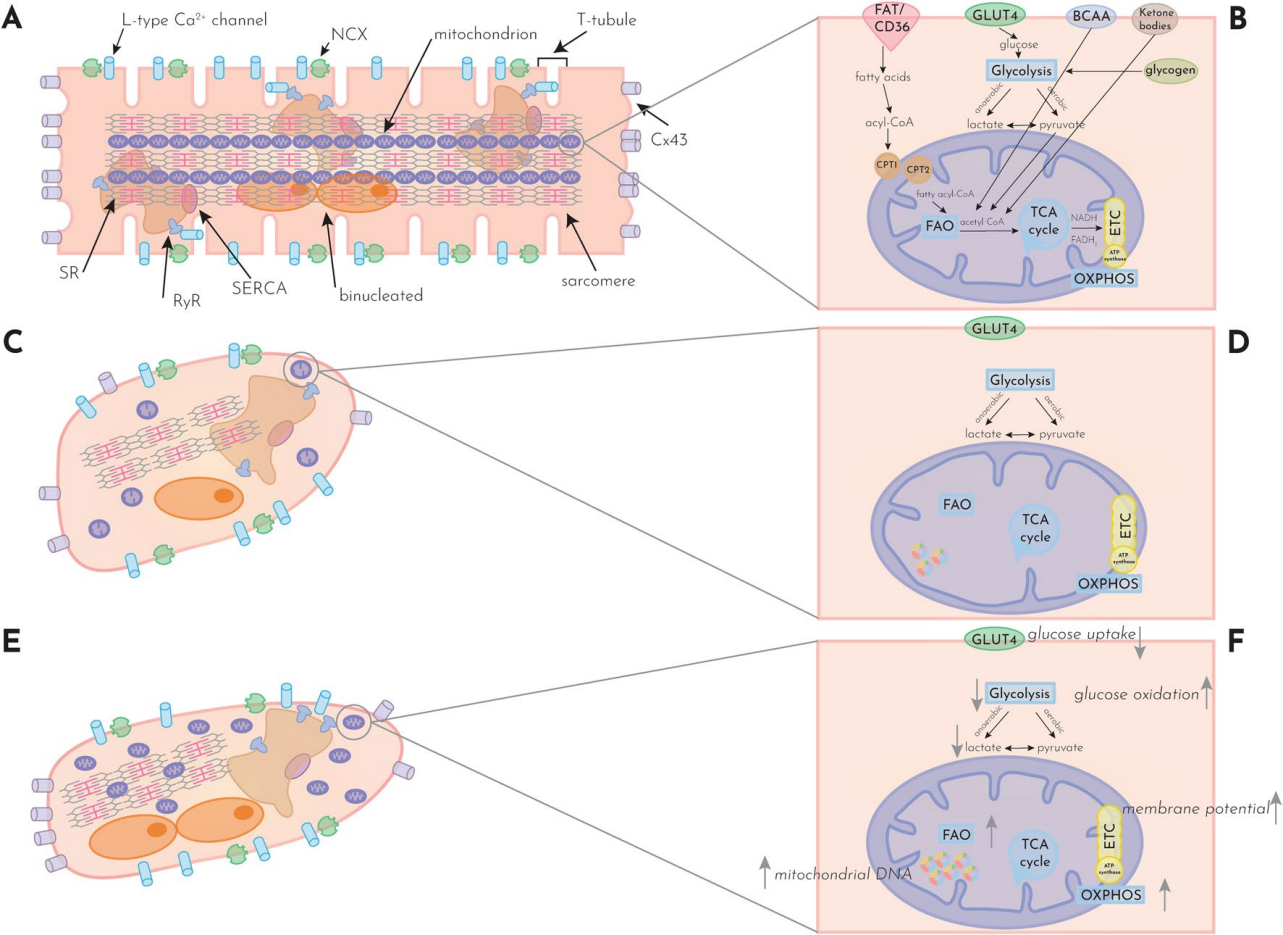
The adult cardiomyocytes versus immature iPSC-CMs and matured iPSC-CMs. Comparison of the structure and metabolism of adult cardiomyocytes (**A**, **B**), immature iPSC-CMs (**C**, **D**) and matured iPSC-CMs (**E**, **F**). **A** Adult cardiomyocytes are elongated, with sarcomeres organized in myofibrils. Mitochondria are aligned with the myofibrils in the horizontal direction of the cardiomyocyte. L-type Ca^2+^ channels are present on the membrane and in the T-tubules, in close proximity to the RyRs to ensure fast calcium signal transduction. Calcium can be extruded via the NCX. **B** Schematic overview of substrate use in the adult cardiomyocyte. Fatty acids enter from the blood via the FAT/CD36 in the cardiomyocyte. After conversion to acyl-CoA, transport into the mitochondria takes place via CPT1 and CPT2 to enter the matrix. Fatty-acyl-CoA enter FAO to yield acetyl CoA, which can enter the TCA cycle. Glucose enters from the blood via the GLUT4, or is derived from glycogen storage in the cardiomyocyte. Glucose enters glycolysis to yield either lactate or pyruvate, which can then cross the mitochondrial membrane. The end product acetyl CoA can enter the TCA cycle. The by-products of the TCA cycle, NADH and FADH_2_ deliver electrons to the ETC to power ATP production at the ATP synthase. **C** iPSC cardiomyocytes are smaller and rounder compared to adult cardiomyocytes, with a single nucleus. Sarcomeres are organized, but not well aligned. L-type Ca^2+^ channels are present, but not in close proximity to RyR. Mitochondria are round with less cristae and are more often perinuclear than peri-sarcomeric. **D** Immature iPSC-CMs demonstrate the main processes in cardiac metabolism. However, iPSC-CMs mainly rely on glycolysis in contrast to FAO for energy production. **E** Matured iPSC-CMs become more elongated, with binucleation, aligned sarcomeres, higher SR maturity (more RyRs) and more mitochondria. **F** Mitochondria increase in mass and change from surrounding the nucleus to surrounding the sarcomeres. Glucose uptake decreases in the matured iPSC-CMs, as well as overall glycolysis, lactate production and glucose consumption (not depicted). Glucose oxidation, glycolytic capacity and glycolytic reserve increase and FAO and oxidative phosphorylation become more pronounced in matured iPSC-CMs. Mitochondrial DNA increases and mitochondrial membrane potential becomes higher. To emphasize the changes that occur during maturation in the iPSC-CMs, only the glucose transporter, and not other substrate transporters, was depicted in **D**, **F**. For the same reason, the presence of mitochondrial DNA was omitted on purpose in **B**. *BCAA* branched chain amino acids; *Ca*^*2*+^ calcium; *CPT1/2* carnitine palmitoyltransferase I/II; *Cx43* connexin 43; ETC electron transport chain; *FAO* fatty acid oxidation; *FAT/CD36* fatty acid translocase/cluster of differentiation 36; *GLUT4* glucose transporter type 4; *iPSC-CMs* induced pluripotent stem cell-derived cardiomyocytes; *NCX* sodium–calcium exchanger; *OXPHOS* oxidative phosphorylation; *RyR* ryanodine receptor; *SERCA* sarco/endoplasmic reticulum Ca^2+^ ATPase; *SR* sarcoplasmic reticulum; *TCA cycle* tricarboxylic acid cycle; *T-tubule* transverse tubules

The cardiomyocyte contracts in response to an electrical signal in a process called excitation–contraction coupling. After depolarization of the cardiomyocyte, L-type calcium channels open to allow an influx of calcium [61]. The calcium that enters the cell stimulates ryanodine receptors (RyRs) on the sarcoplasmic reticulum (SR) to release more calcium into the cell [5] (Fig. 1A). In response to increasing calcium concentration, calcium binds to troponin C (cTnC), causing a conformational change of troponin–tropomyosin complex, which thereby uncovers myosin-binding sites on actin. The interaction of myosin heads and actin and myosin’s subsequent power stroke causes sliding of the myofilaments and muscle contraction. Cardiac myosin-binding protein C limits the mobility of myosin heads acting as a brake on contraction [75]. During relaxation, calcium is extruded via the sodium–calcium exchanger (NCX) or pumped back into the SR by the sarco-endoplasmic reticulum Ca^2+^ ATPase (SERCA), leaving less calcium available to bind to cTnC [5]. The process of excitation–contraction coupling requires a high energy supply needed to fuel ion pumps, the calcium handling machinery and contraction and relaxation of the cardiomyocyte.

The heart metabolizes a variety of substrates to generate adenosine triphosphate (ATP) (Fig. 1B), predominantly fatty acids (FAs), carbohydrates, and to a lesser extent branched chain amino acids and ketone bodies [51, 60]. FAs come from the plasma as free FAs bound to albumin or from stored triglycerides and are broken down for energy production by β-oxidation (fatty acid oxidation (FAO)) [81]. FAs move into the cell through the transporter fatty acid translocase/cluster of differentiation 36 (FAT/CD36), are esterified into acyl-CoA in the cytosol, and subsequently enter the mitochondria using the carnitine shuttle comprised of carnitine palmitoyltransferase 1 (CPT1), carnitine-acylcarnitine translocase (CACT) and carnitine palmitoyltransferase 2 (CPT2) [73]. Exogenous glucose enters the cell via the insulin-dependent GLUT4-transporter and, to a lesser extent, the GLUT-1 transporter or is derived from stored glycogen. Glucose enters glycolysis in the cytoplasm to produce pyruvate, which subsequently enters the tricarboxylic acid (TCA) cycle for further oxidation (aerobic) or conversion to lactate (anaerobic). All substrates yield acetyl-CoA which enters the Krebs cycle, also known as the TCA cycle [51]. FAO, glycolysis, pyruvate oxidation, lactate oxidation and the TCA cycle yield NADH, and FADH_2_ is obtained in FAO and the TCA cycle. These reductive equivalents deliver electrons for the electron transport chain (ETC), which is a series of five protein complexes in the inner membrane of mitochondria consisting of complexes I–IV and the ATP synthase, to power the generation of ATP via oxidative phosphorylation [7].

In the heart, 95% of all ATP is produced via oxidative phosphorylation, while the remaining 5% is derived from glycolysis. The healthy heart uses ~ 40–60% FAs for ATP production, 20–40% glucose from oxidation, 10–15% ketones, 10–15% lactate, 2–8% glucose from glycolysis and < 2% branched chain amino acids [51]. The contribution of any substrate to ATP production depends on the energy demand of the heart, substrate availability, hormone levels and oxygen levels [67]. The healthy heart is able to switch rapidly between substrates and alter metabolism accordingly to maintain contractile function and energetic homeostasis, a unique characteristic referred to as ‘metabolic flexibility’ [80].

## Metabolic characteristics of maturing iPSC-CMs

The structural, electrophysiological, contractile and metabolic characteristics of iPSC-CMs are underdeveloped in comparison with adult cardiomyocytes (Fig. 1A–D) [17, 62, 87]. This roadblock has been widely addressed in recent research, and the main differences between native adult human cardiomyocytes and iPSC-CMs have been well documented [20, 40, 72, 76]. For example, the immaturity of iPSC-CMs is reflected by their metabolic phenotype where iPSC-CMs rely on aerobic glycolysis for their ATP production with little contribution of oxidative phosphorylation [43]. In addition, it has been widely reported that iPSC-CMs resemble fetal or neonatal cardiomyocytes, lacking certain gene expression profiles and pathways associated with the adult phenotype [92, 94]. The immature state achieved by current methods limits the applicability of iPSC-CMs for preclinical and clinical purposes, such as disease modeling, drug safety testing and regenerative medicine [40].

Considerable attention has been given to this issue, and several strategies have been developed for maturing iPSC-CMs, including prolonged culture, culture in 3D, electrical pacing, culture with non-cardiomyocytes, and adjusting media composition by addition of fatty acids and supplementation of hormones. Culturing iPSC-CMs for up to one year showed increased maturation evident from ultrastructural sarcomeric changes that resemble mature cardiomyocytes [39]. In addition, culturing iPSC-CMs in 3D structures such as engineered heart tissue resulted in higher functional and structural maturity [13, 78], as did gradually increasing electrical pacing [34, 71, 72]. When iPSC-CMs were cultured with iPSC-derived cardiac fibroblasts and iPSC-derived endothelial cells in 3D microtissues, notable functional, metabolic and structural maturation was recorded [26]. One of the other promising maturation strategies is keeping iPSC-CMs in non-standard media, with both hormones [64, 92, 94] and fatty acids [21, 35]. These approaches are directly informed by the native development that cardiomyocytes undergo in the neonatal stage, providing the substrates necessary to mature their metabolic state.

While maturation studies mostly focused on the morphology, structure and electrophysiology of developing cardiomyocytes, increased emphasis is given to the metabolism of iPSC-CMs. Here, we review specifically how the metabolism of iPSC-CMs changes upon the use of strategies to improve maturity of iPSC-CMs. Our search strategy consisted of entering the following term in the PubMed (MEDLINE) database: ‘iPSC cardiomyocytes maturation metabolism’ and screening the resulting 243 hits for the words ‘glycolysis,’ ‘fatty acid oxidation,’ ‘OCR,’ ‘ATP,’ ‘respiratory’ and ‘mitochondria.’ Out of the articles containing at least one of these words, the ones describing an approach to improve iPSC-CM metabolic maturity were selected and used for this review. Additional articles for the review were collected from the reference lists of the articles selected in the initial search, leading to a total of 23 studies. Additional file 1: Table S1 provides an overview of the studies addressing the metabolic maturation of iPSC-CMs upon the use of maturation strategies. The quantitative data provided in this table for each of the parameters examined were retrieved from direct communication with the authors of the respective studies, and when no response was obtained from the corresponding author, the values were scored from the bar graphs in the figures of the relevant articles by two independent observers. To check the accuracy of our scored values, we also scored values for six papers (with 49 metabolic characteristics in total) for which we received original data. This comparison yielded Pearson correlation coefficients of 0.99 for both maturation and HCM and yielded an average variation between − 3.2% to 2.8% (maturation) and − 3.0% to 3.4% (HCM) between scored and original data. Extreme values were additionally examined by a third observer for inclusion. Scored values were averaged, and the average is depicted by ~ in the table. Excluded values were marked by an asterisk and reported in “Appendix”.

### Gene expression

Gene expression levels were reported in 18 out of 23 studies that examine metabolism in maturing iPSC-CMs. Expression levels of genes that regulate cardiac metabolism such as *PDK4, CD36, PPARA*, *ATP5*, *LPL*, *ACAT1*, *DGAT1* and *CPT1A/1B* increase in iPSC-CMs when they are subjected to maturation strategies such as prolonged culture [4, 13, 18], 3D tissues [26] and fatty acid supplementation [14, 21, 24, 35, 52, 70, 93, 95]. In addition, the expression of mitochondrial biogenesis-related genes including *PPARGC1A* and *ESRRA* was upregulated when iPSC-CMs were matured by prolonged culture [13] and fatty acid supplementation [70]. Expression of genes related to FAO, for instance *SCD*, *PPARD* and *ACADVL*, also increased in studies that applied fatty acid supplementation [14, 21, 70, 93, 95]. At the same time, glycolysis-related genes such as *ALDOA*, *HK1*, *HK2*, *PGK1*, *GAPDH* and *LDHA* were downregulated using prolonged culture [13], fatty acid supplementation [21] and inhibition of mTOR, a regulator of cellular metabolism and growth [23]. These findings are in accordance with the developmental switch from glycolysis to FAO which occurs in maturing cardiomyocytes [36]. In most of the above studies, quantitative reverse transcription polymerase chain reaction (RT-qPCR) and RNA sequencing (RNA-seq) were used to investigate gene expression changes. Correia et al. [13] performed transcriptome profiling and pathway analysis to show differences in enrichment of various pathways. Ulmer and colleagues compared the proteomic profile and mitochondrial proteome of iPSC-CMs with and without maturation protocol with non-failing human adult cardiomyocytes and concluded that the proteomic profiles of matured iPSC-CMs are more similar to that of the adult human heart than non-matured iPSC-CMs [87]. Such in-depth analyses can complement RNA-seq and RT-qPCR measurements by providing additional information about patterns of gene expression, protein levels and changes in entire metabolic pathways and thus deliver a more comprehensive overview of the metabolism of iPSC-CMs with respect to human cardiac tissue. However, gene expression or protein levels do not necessarily translate into functional improvement and caution should be taken when interpreting such data.

### Mitochondria

To enable the developmental transition toward FAO as the main energy source in cardiomyocytes, mitochondria in maturing cells grow in size and become elongated, their membrane potential increases, and they move from the perinuclear position toward the sarcomeres and acquire more developed cristae [38, 83]. Mitochondrial elongation and cristae development have been observed upon applying various maturation strategies including 3D culture [26, 71] and fatty acid supplementation [21, 70, 95]. Similarly, increases in mitochondrial numbers and size upon iPSC-CM maturation have been recorded, reflected after fatty acid supplementation by an elevated intensity of mitochondrial staining [21, 36] as well as a higher amount of mitochondrial DNA (mtDNA) [21, 36, 95]. Determining the ratio between mtDNA and genomic DNA (gDNA)/nuclear DNA (nDNA) can be used to assess metabolic maturation—mtDNA normalized to gDNA provides information on mitochondrial content in the cell which increases during maturation, though data scatter in iPSC-CM studies is large [86]. In addition, more peri-sarcomeric mitochondria and fewer perinuclear mitochondria have been detected in two maturation studies that employed fatty acid supplementation [21, 24]. Prolonged culture resulted in increased fusion of mitochondria in maturing iPSC-CMs and increased mitochondrial networks [4, 70], an increased number of branches and an increase in the average mitochondrial branch length [18]. In addition, elevated levels of mitochondrial calcium were found, which can promote the activity of calcium-dependent mitochondrial enzymes [18]. Another change that indicates higher functionality of these organelles is the increase in mitochondrial membrane potential (MMP or ΔΨm), as measured by mitochondrial dyes such as JC-1, tetramethylrhodamine (m)ethyl ester (TMRE/M) and MitoView™ in iPSC-CMs matured by prolonged culture or fatty acid supplementation [18, 70, 95]. MMP is a useful indicator of mitochondrial maturation since it gives a measure of the functionality of the ETC and oxidative phosphorylation. Dyes that stain mitochondrial biogenesis or mitochondrial mass (e.g., MitoTracker™ Green) may be used in combination with a dye sensitive to MMP (e.g., TMRM) to derive a relative quantification of MMP independent of total mitochondrial mass [65, 70].

### Respiration capacity and rate

The majority of studies (21 out of 23) use the Seahorse XF assay to study the rate and capacity of respiration (Additional file 1: Figure S1A-B); this approach provides data about basal respiration, maximal respiration and spare respiratory capacity by assessing the oxygen consumption rate (OCR). By measuring respiration and adding several inhibitors of the ETC, the proportion of oxidative phosphorylation can be deducted as an estimation of the oxidative capacity of the cardiomyocyte. A higher OCR indicates that the ETC uses more oxygen to produce more ATP, and an increased ATP production is characteristic of metabolic maturity. An increased spare respiratory capacity indicates a higher reserve to meet increases in energy demand, which is another characteristic of metabolic maturity. Of note, Seahorse assays also indirectly measure proton leak, indicating the uncoupling between ADP phosphorylation and substrate oxidation. An increase in proton leak might indicate oxidative stress, but can also be a physiological response, e.g., due to increased expression of uncoupling proteins as a result of maturation strategies [37]. Maturation approaches, including prolonged culture, 3D culture and fatty acid supplementation, result in notable increases in respiration parameters such as basal respiration (12 out of 23 studies) [4, 14, 21, 24, 26, 35, 36, 57, 71, 92, 94–96], maximal respiration (14 out of 23 studies) [4, 9, 21, 23, 24, 26, 35], Junjun [48, 57, 70, 71, 92–95] and spare respiratory capacity (10 out of 23 studies) [4, 21, 23, 24, 26, 35, 70, 71, 92, 94, 96]. Increased proton leak was reported in three studies that employed prolonged culture [4], fatty acid supplementation [24] and 3D culture [26]. Decreased proton leak was reported in one study that applied fatty acid supplementation [95]. Moreover, higher expression of genes encoding ETC proteins has been documented in matured iPSC-CMs after prolonged culture in 3D [87], as well as elevated abundance of the ETC proteins themselves after prolonged culture in 2D [70].

### Glucose metabolism

Glucose metabolism can be assessed in various ways, for example, by measuring the extracellular acidification rate (ECAR) using Seahorse technology [4, 18, 21, 23, 26, 71, 96]. From the ECAR, several parameters can be derived such as glycolytic rate, glycolytic capacity and glycolytic reserve as an estimation of glycolysis (Additional file 1: Figure S1C). Other methods to measure glucose metabolism in iPSC-CMs include radioactive isotope tracing (Additional file 1: Figure S2) [18, 52, 87] and stable isotope tracing [14]. Some iPSC-CM maturation studies report an increase in ECAR after prolonged culture [4], 3D culture [26, 71] and fatty acid supplementation [21, 23], while others report the opposite after prolonged culture or fatty acid supplementation [13, 14] or no changes in ECAR after 3D culture or fatty acid supplementation [18, 96]. Glucose oxidation, calculated from metabolic flux or determined by ^14^CO_2_-capture, was heightened after 3D culture [13, 87] and fatty acid supplementation [52]. On the other hand, glucose uptake and ATP production from anaerobic glycolysis in iPSC-CMs were reported to be decreased using 3D culture [13, 87], as well as the amount of glycogen deposits and glucose consumption itself [87].

Additional glycolytic parameters have been used for evaluating glucose metabolism in iPSC-CMs. Hexokinase activity was reported to be lowered after 3D culture [87], prolonged culture [70] and fatty acid supplementation, as measured by hexokinase assays [36, 95]. In addition, pyruvate dehydrogenase activity was upregulated after fatty acid supplementation [14], indicating reduced anaerobic metabolism. Furthermore, lactate secretion was markedly diminished prolonged culture [13], after 3D culture [26, 36, 87] and fatty acid supplementation [14, 36, 95]. The decrease in glycolytic enzymes as well as the decrease in lactate secretion indicate a shift away from glucose metabolism and can therefore be considered as indicators of metabolic maturation. Various techniques were used for lactate measurements, including blood gas analysis [21, 87], isotope tracing [87], lactate assay kits [36, 95], nuclear magnetic resonance [26] and biochemistry analyzers [13, 14]. The ratio of lactate production to glucose consumption was also reduced in matured iPSC-CMs after fatty acid supplementation [21], again implying a transition from anaerobic to aerobic glucose metabolism. Culture of iPSC-CMs in glucose-free media enriched with FA and galactose highly upregulated pyruvate dehydrogenase activity, shifted cytosolic pyruvate to the TCA cycle and away from lactate production, and also markedly increased the contribution of the pentose phosphate pathway to total glycolytic fluxes [14]. Taken together, these findings indicate that, even though the maturing iPSC-CMs may have an increased capacity for metabolizing glucose, the simultaneous development in FA metabolism capabilities reduces the need for ATP production through glycolysis—hence more mature iPSC-CMs show a reduction in glucose uptake, glycogen storage, lactate production, hexokinase activity and proportion of glycolysis-related ATP production.

### Fatty acid metabolism

The degree of FAO in iPSC-CMs can be estimated using the Seahorse assay (Additional file 1: Figure S1D) [18, 24, 35, 95], free fatty acid uptake assay [21], and fatty acid flux analysis using stable or radioactive isotopes [14, 52, 87]. Although four studies evaluate FAO by using etomoxir to inhibit FAO and then measure OCR using Seahorse, it has recently been reported that etomoxir has unspecific off-target effects that may influence the measurement [53]. In addition, the OCR quantified in the assay may not account for the entire proportion of FAO [53]. For these reasons, Seahorse should be regarded only as a means to estimate FAO. Isotope tracing revealed an upregulated FAO [52] and an elevated contribution of fatty acids to TCA cycle activity in iPSC-CMs exposed to fatty acids [14]. Increased enrichment in pathways related to oxidation of branched and very long-chain FAs has been recorded upon prolonged culture [9]. Enrichment analysis of 3D-cultured iPSC-CMs demonstrated upregulation of the TCA cycle and a reduction in FA synthesis [13, 87]. Fatty acid supplementation also stimulated FA uptake, as determined by a free FA uptake assay [21] and prolonged culture increased L-carnitine levels [70]. Notably, maturing iPSC-CMs using 3D tissue preparations increased the relative contribution of FAO to ATP production from 40 to 65%, which is strikingly close to the percentage of ATP that is produced by the same process in an adult cardiomyocyte in vivo [87]*.*

### ATP production

As the heart grows and matures, cardiomyocytes need to synthetize increasing amounts of ATP to support sustained energetically expensive contractions and protein turnover. For this reason, it is crucial to examine whether maturation strategies induce an upregulation in ATP production arising from increased oxidative phosphorylation in the cell. Multiple methods for evaluating ATP synthesis directly and indirectly in iPSC-CMs are available, such as Seahorse [4, 21, 35], calculation from flux analyses [13, 14, 87], cell viability assays [36] and luciferase kits [70, 95]. Eleven out of 23 studies provide evidence that this is indeed the case-different maturation strategies have been successful in stimulating ATP production, such as prolonged culture [4], 3D culture [13, 26, 87], hormonal supplementation [57] and fatty acid supplementation [14, 21, 24, 35, 95, 96].

In short, maturation protocols promote the metabolic maturation of iPSC-CMs, which result in improved mitochondrial structure and function, a decrease in glycolytic activity, an increase in FAO and an increase in ATP production. Prolonged culture, 3D culture and fatty acid supplementation were the most commonly used protocols that achieved metabolic maturation in iPSC-CMs, which will be elaborated further in the discussion.

## Metabolic characteristics of HCM iPSC-CMs

iPSC-CMs are increasingly used to model HCM (a systematic overview can be found in [19]), where the main focus thus far has been on modeling cardiomyocyte hypertrophy and abnormalities in contractility, electrophysiology and calcium handling [19, 47]. Although metabolic dysfunction is a hallmark of HCM pathology, it has received less attention than other cardiomyocyte alterations. To create an overview of the metabolic characteristics that have been studied in HCM iPSC-CM models, we entered the term ‘hiPSC cardiomyocytes hypertrophic cardiomyopathy’ into the PubMed (MEDLINE) database and selected the resulting 86 hits for the words ‘glycolysis,’ ‘fatty acid oxidation,’ ‘OCR,’ ‘ATP,’ ‘respiratory’ and ‘mitochondria.’ We not only included iPSC-CM models on HCM specifically, but also included metabolic diseases with a cardiomyopathy phenotype, resulting in a total of 13 studies. An overview of all quantitative metabolic measurements is presented in Additional file 1: Table S2.

### Patient and study characteristics

Studies on iPSC-CM models of HCM mostly involve sarcomere mutations, with *MYH7* being the most prevalent (6/13), coding for myosin heavy chain-β, followed by *MYBPC3* (2/13), coding for cardiac myosin-binding protein C, *ACTC1* (2/13), coding for α-actin and *TNNT2* (1/13), encoding cardiac troponin T. Metabolic disorders with the HCM phenotype included several storage-related diseases, such as the lysosomal storage disease Fabry disease, caused by a mutation in the α-galactosidase A gene [11]. One study investigated Danon disease, a multisystem disorder with skeletal and cardiac muscle involvement, caused by a mutation in the lysosomal associated membrane protein-2 (*LAMP2*) gene [32]. Two other studies focused on protein kinase AMP-activated non-catalytic subunit gamma-2 (*PRKAG2*) cardiac syndrome, a disease characterized by left ventricular hypertrophy, ventricular pre-excitation and glycogen accumulation [33, 97]. *PRKAG2* encodes one of the three subunits of 5’AMP-activated protein kinase (AMPK), which senses cellular energy and nutrient levels and accordingly regulates several cellular functions to maintain energy homeostasis [31]. Two studies included mitochondrial disorders, of which one study focused on the primary mitochondrial disease Barth syndrome [89] and one study reported on a mitochondrial cardiomyopathy (mutation in *MT-RNR2*) [49]. All studies included iPSC-CMs from one to three individuals, where the studies with multiple patients included individuals with differences in severity of disease or in type of mutation. The lines that were used in the studies were derived from patients (8 out of 13 studies) [11, 32, 33] [49, 68, 69, 89, 97] and/or manufactured by introducing a known pathogenic gene variant into a wild-type hiPSC line (6 out of 13 studies) [8, 12, 33, 41, 59, 84]. Most of the studies used isogenic controls (8 out of 13 studies) [8, 12, 33, 41, 59, 68, 84, 89]. The other studies relied on one to three healthy controls with varying degrees of age- and sex-matching and differences in completeness of reporting information.

### Mitochondria

Sarcomere mutations are hypothesized to cause inefficient contraction in the cardiomyocyte, leading to a high energy demand at the myosin ATPase and increasing the workload of mitochondria [2]. Mitochondrial biogenesis (mass) increased in *MYH7* [12, 84] and *MYBPC3* iPSC-CM lines [12]. However, mtDNA/gDNA ratio was similar between diseased and control lines in other studies on *MYH7* [41, 59]. For the metabolic disorders, Danon disease iPSC-CMs demonstrated a higher number of abnormal mitochondria, indicated by Parkin- and p62-positive staining, as well as a decrease in mitochondrial membrane potential [32]. No obvious mitochondrial morphological differences were found in *PRKAG2*-mutated iPSC-CMs [97], while mitochondrial content increased in a different study on *PRKAG2* [33]. For the mitochondrial disorders, mitochondrial membrane potential increased in Barth syndrome iPSC-CMs [89]. Mitochondria were round and immature with underdeveloped cristae in *MT-RNR2*-mutated iPSC-CMs. Mitochondrial content and mtDNA copy number increased, while mitochondrial membrane potential decreased [49]. An increase in mitochondrial properties suggests increased mitochondrial work to comply with the elevated energy demand, while a reduction in mitochondrial membrane potential reflects the limited involvement of the ETC and oxidative phosphorylation in ATP production.

### Respiration capacity and rate

The increasing workload of mitochondria in HCM is initially expected to boost respiration rates in the cardiomyocyte as a compensatory mechanism, before decreasing as HCM progresses [8]. Respiration capacity and rates were measured in most studies (10 out of 13 studies) and were all assessed by Seahorse XF assays. Basal and maximal OCR increased in iPSC-CM lines with mutations in *ACTC1* [8, 41], *TNNT2* [68] and *MYH7* [8, 41, 59, 68, 84]. Basal and maximal OCR increased with mutation load in *MYH7*-mutated lines, with the lowest OCR in the wild-type line and the highest OCR in the homozygous mutated line [59]. For the metabolic disorders, respiratory activity decreased in Fabry and Danon disease [11, 32]. In Fabry disease iPSC-CMs, in vitro enzyme replacement therapy restored the deficient α-galactosidase A, improved enzyme activity and decreased glycosphingolipid accumulation, but failed to recover the basal OCR [11]. Introducing the healthy copy of *LAMP-2B*, the most abundant cardiac isoform of *LAMP-2* which is deficient in Danon disease, only partially restored maximal respiratory capacity in diseased iPSC-CMs [32]. For PRKAG2 cardiac syndrome, basal and maximal respiration increased in iPSC-CM lines [33, 97]. Maximal respiration and respiration capacity were higher in *PRKAG2*-mutated iPSC-CMs derived from a severely affected patient compared to a mildly affected patient [97]. Respiration rates were also altered in primary mitochondrial disorders, with increased basal respiration but decreased reserve capacity in Barth syndrome iPSC-CMs [89]. Interference of nucleoside-modified messenger RNA and small molecules mitigated the mitochondrial dysfunction and restored basal OCR, but failed to recover maximal respiratory capacity [89]. The metabolic disorders show that correcting the phenotype e.g., by introducing the healthy copy of the affected gene, does not sufficiently mitigate the metabolic aberrances in the diseased cardiomyocyte.

### Glucose metabolism

When HCM progresses toward heart failure, the energy metabolism shifts from FAO to glycolysis for ATP production, increasing the resemblance to fetal metabolism where carbohydrates are the main source of energy production [50, 82]. *MYH7*-mutated iPSC-CMs demonstrated upregulation of intermediates in the glycolytic pathway [84]. *PRKAG2*-mutated iPSC-CMs showed an increase in glucose uptake and upregulation of glycolysis intermediate glucose-6-phosphate. However, downstream glycolytic intermediates were reduced and activity of glycolytic enzymes decreased. Levels of glycogen precursors increased accompanied by augmented intracellular glycogen [33]. In another study on *PRKAG2*, mutated iPSC-CMs demonstrated increased glycogen associated vacuoles, accompanied by a slight increase in glycogen content in both patient lines. However, increased glycogen synthase activity was only found in the severely affected patient cell line [97].

### FAO

In progressing HCM, the proportion of FAO used for ATP production is expected to decrease in diseased cardiomyocytes [50]. An increase in TCA intermediates was argued to be representative for active FAO in *MYH7*-mutated iPSC-CMs [84]. Measuring absolute OCR revealed less FAO in Fabry disease iPSC-CMs, which was associated with a decrease in the expression of FA metabolism-associated genes and an increase in glucose metabolism genes [11]. *PRKAG2*-mutated iPSC-CMs with a gain of function in AMPK, known for increasing FAO, presented an increase in FAO intermediates together with an increase in expression of FAO genes [33].

### ATP production

ATP production increased in *ACTC1* and *MYH7*-mutated iPSC-CM lines [41, 59]. Additionally, *MYH7*-mutated iPSC-CM lines were observed to have an increased ADP/ATP ratio, indicating a marker of metabolic stress [12]. Also, the ratio between ATP and phosphocreatine, used to regenerate ATP at the myosin ATPase, was decreased in *MYH7*-mutated iPSC-CMs [84]. However, absolute ATP levels did not decrease in the diseased iPSC-CM lines, similar to findings in other experimental models where ATP levels remained constant [77]. For the metabolic diseases, the ratio between ATP content and production (ATP turnover) decreased in Danon disease iPSC-CMs, suggesting a lower rate of ATP production [32]. ATP-linked respiration or ATP turnover was elevated in iPSC-CMs from a severely affected patient with a *PRKAG2* mutation, while this balance was not changed in a mildly affected patient [97]. For the primary mitochondrial disorders, the ATP/ADP ratio was decreased in iPSC-CMs from HCM patients carrying the mitochondrial *MT-RNR2* mutation [49]. Culturing iPSC-CMs in galactose and thereby limiting ATP production via glycolysis yielded a lower ATP content in Barth syndrome iPSC-CMs compared to healthy iPSC-CMs [89].

### Oxidative stress

Oxidative stress is a common hallmark of failing cardiomyocytes due to the increased mitochondrial workload [6], which can be observed both in HCM and in metabolic diseases with an HCM phenotype. The production of reactive oxygen species (ROS) was increased in *MYH7*-mutated iPSC-CMs and could be suppressed by gene ablation which led to improved twitch force [12]. In other studies on *MYH7*, ROS levels did not differ significantly to control [41, 59]. The NAD^+^/NADH ratio was elevated in *MYH7*-mutated iPSC-CMs, indicating a shift in redox balance [84]. Oxidative stress was also quantified by assessing ROS production and myeloperoxidase (MPO) activity in *MYBPC3*-mutated iPSC-CMs. MPO is predominantly associated with inflammation, but has also been found in diseased hypertrophied human hearts [69]. *MYBPC3*-mutated iPSC-CMs showed higher MPO protein levels, which were associated with increased chlorination and peroxidation. Inhibiting MPO improved relaxation and relieved calcium signaling defects in the affected CMs [69]. Excessive ROS production was also observed in Barth syndrome iPSC-CMs, where scavenging of ROS by small molecules ameliorated the mitochondrial dysfunction [89].

## Discussion

Studying mechanisms of disease in vitro has been empowered by the development of iPSC-CMs that provide a virtually limitless supply of cells while retaining the genetic signature of the individual they are derived from. In this study, we compiled the iPSC-CM studies that examined the effect of maturation approaches on metabolism and studies that included metabolic measurements in iPSC-CM lines with a mutation that causes HCM or an HCM phenotype.

**Fig. 2:**
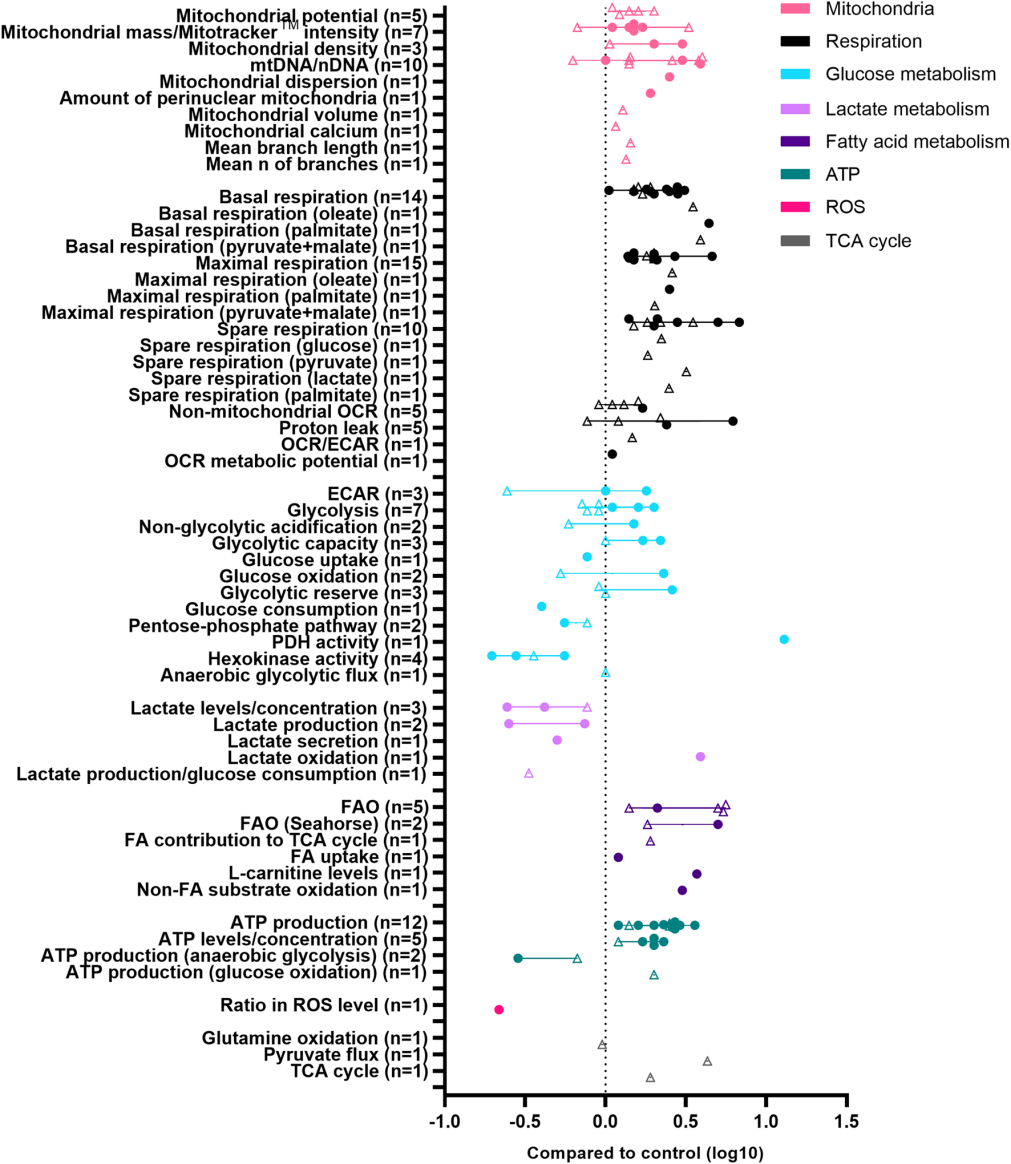
Metabolic characteristics of maturing iPSC-CMs. Overview of maturation-induced changes in metabolic parameters measured in iPSC-CMs and reported in the literature. Each data point represents a log10 fold change compared to control of a single parameter reported in a publication. The quantitative data were obtained from the text, figures or supplemental data of each publication. When the exact value could not be obtained from the publication or its supplements, the quantitative data were retrieved from direct communication with the corresponding author of the respective publication. When no response was received from the authors, the fold change was scored from the bar graphs in the respective study by two reviewers independently, and the mean of the independent assessments was entered as a data point. Circles represent original data, open triangles represent scored data. *ATP* adenosine triphosphate; *DHE* dihydroethidium; *ROS* reactive oxygen species

For the metabolic profile of maturing iPSC-CMs, a clear pattern can be observed from the reported metabolic parameters (Fig. 2). As explained above, mitochondria in maturing iPSC-CMs increase in mass and abundance, contain a higher mtDNA/gDNA ratio, and have a higher membrane potential, pointing to increased ETC activity and more FAO. Respiratory parameters also increase in more mature iPSC-CMs compared to non-matured cells, including increased basal respiration, maximal respiration and spare respiratory capacity. Lactate levels are lowered by maturation, which indicates a reduction in anaerobic glycolysis and a transition toward oxidative metabolism. This conclusion is supported by the elevated FAO and ATP production parameters reported across studies. However, the characteristics of glucose metabolism in maturing iPSC-CMs are less clear. As seen in Fig. 2, parameters such as ECAR and ATP production from anaerobic glycolysis both increase and decrease in different studies. While glycolysis, glucose uptake, glucose consumption and hexokinase activity reduce upon maturation, glycolytic capacity, glucose oxidation, and reserve parameters increase upon maturation. Such findings can indicate a preference for FAO in more mature iPSC-CMs, with an accompanying increase in their ability to metabolize glucose, switch between available substrates and response to changing energy demands. A schematic overview of the metabolism in matured compared to non-matured iPSC-CMs is provided in Fig. 1C–F.

The metabolic profile of matured iPSC-CMs described above is in accordance with the developmental changes that occur in adult human cardiomyocytes which produce the majority of their ATP through FAO. Fetal cardiomyocytes rely mostly on glycolysis for ATP production since the fetal heart experiences a low work load and grows in a relatively hypoxic environment in the presence of insulin [3, 27]. After birth, the cardiomyocytes hypertrophy and experience more mechanical load and require more energy. The newborn diet, consisting of carbohydrate and lipid-rich milk, the increased oxygen content in the heart and reduction in insulin concentration all lead to a switch to FAO as the primary source of energy for the heart, stimulating mitochondrial growth, as well as the development of cristae and mitochondrial networks [3]. However, the heart still retains the ability to use a variety of substrates for energy, especially in states of increased ATP demand [27, 46]. Based on the results compiled in this study, iPSC-CMs seem to undergo similar metabolic changes, but their metabolism is still not fully mature. For example, mitochondria in native adult cardiomyocytes occupy around 30% of the entire cell volume [74], in contrast to ~ 7–10% in iPSC-CMs even after maturation [92, 94]. Mitochondrial density increased to 30% in only one study we included in our overview [71]. Also, the abundance of mitochondrial proteins and DNA content do not attain adult human heart values, which were, respectively, 1.3 fold and 1.5 fold lower in iPSC-CM engineered heart tissues compared to non-failing adult heart tissue [87]. Furthermore, iPSC-CMs still rely more on anaerobic glycolysis and lactate oxidation than on FAO for ATP production [86]. Nevertheless, the increase in metabolic properties seen in iPSC-CM maturation studies is a compelling reason to explore even further the most effective ways for maturing iPSC-CMs.

After screening our included studies, we identified a couple of pathways that might be involved in metabolic maturation. Metabolic matured iPSC-CMs display a downregulation of the PI3K/AKT/insulin pathway [14], and inhibiting its downstream targets HIF-1α [36] and mTOR [23] also improved the metabolism of iPSC-CMs. These pathways are involved in glycolysis, and downregulation might promote the switch from glycolysis to FAO for energy production. In turn, increasing the concentration of fatty acids in the culture medium upregulates AMPK, ERK and p38 MAPK pathways [93]. The PPAR pathway, a well-known regulator of fatty acid metabolism, also enriched upon maturation using fatty acid supplementation [14, 22, 52]. The cAMP signaling pathway, a regulator of excitation–contraction coupling, also increased in matured iPSC-CMs after 3D culture and fatty acid supplementation. These signaling pathways all might contribute to governing the metabolic switch from glycolysis to fatty acid metabolism for energy production.

**Fig. 3:**
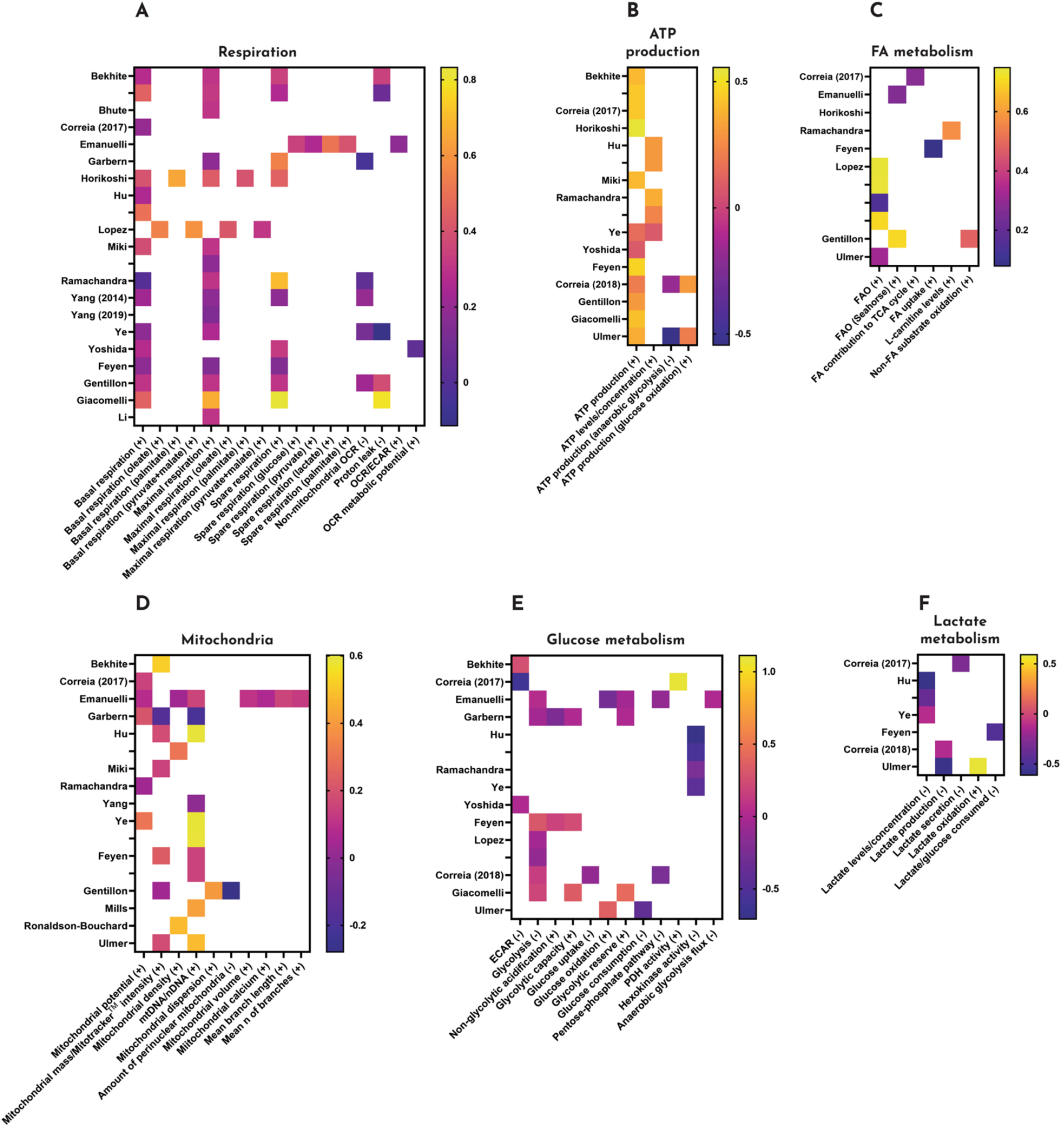
Heatmap of matured metabolism in iPSC-CMs. Overview of the fold changes (after logarithmic transformation) in different metabolic categories to visualize the highest metabolic maturation in iPSC-CMs. **A** Respiration, **B** ATP production, **C** FA metabolism, **D** mitochondria, **E** glucose metabolism, **F** lactate metabolism. The y-axis depicts the different studies indicated by the first author and multiple studies by the same author are indicated by (year) behind the name. Some studies included multiple conditions that were measured separately, indicated by dashes directly under the author name. The x-axis depicts the subcategories within each category. The values that are reported as changed in maturation are indicated by ( +), values that are expected to decrease are indicated by ( −). Studies that did not include measurements in the respective categories were not included in this overview

Based on the maturation studies we reviewed in this study, it is difficult to claim which of the maturation strategies is most effective to improve the metabolism of iPSC-CMs. Since each study measured different metabolic parameters and we gained access to half the source data of all studies (18 out of 36 studies), sound statistical comparisons could not be made. We compared the studies based on the reported fold changes, where we defined the highest metabolic maturation as the highest mean fold increase and/or decrease in most of the selected categories (Fig. 3). The highest metabolic maturation was achieved in 3D tissues subjected to contractile work (increase in ATP production and mitochondrial development, decrease in lactate levels), 3D tissues containing non-cardiomyocytes (increase in respiration, despite the high proton leak) and 3D tissues with active FAO stimulation (increase in FA metabolism). 3D culture has been shown to increase the maturity of iPSC-CMs in terms of structure, electrophysiology and contractility [1]. Supplementing iPSC-CMs with FAs has been shown to increase FAO in the cardiomyocyte [21, 52, 93], possibly contributing to metabolic flexibility. A combination of strategies might therefore prove to be the most effective in achieving metabolic maturation.

Metabolic maturation, in turn, also enhanced the structure, electrophysiology and contraction of iPSC-CMs, possibly linking improved metabolism to physiological maturation [21]. Metabolic maturation might also have some disadvantages, as studies that employed a metabolic maturation protocol warn against using only fatty acids in the culture medium, which might induce fatty acid build-up and cellular toxicity [14]. In addition, depleting the cells of glucose might promote ROS production [21]. In general, both studies plea for a balanced culture medium with appropriate substrate concentrations. Even though the fold changes provide valuable insight into the effect sizes of the different strategies, caution should be taken when interpreting the absolute values that are reported here. Fold changes depend on the quality of the used iPSC-CM lines and could lead to exaggerated effect sizes when the control line is of low quality, as exemplified in “Appendix”. Values could also be influenced by certain experimental conditions. For example, the media that were used for Seahorse experiments contained varying concentrations of glucose/galactose (5–25 mM), while the physiological fasting blood glucose concentration is around 3.5–5.5 mM [30]. Using non-physiological substrate concentrations might cause the cardiomyocyte to favor a certain substrate, which might not be a true reflection of basal cardiac metabolism. Nevertheless, bottlenecks are likely to persist in the field of iPSC-CM maturation and disease modeling. It is highly probable that maturity levels achieved in the native state will not be reached in vitro with the technologies currently available. Furthermore, there are still gaps in the knowledge on cardiomyocyte maturation both in their native environment and in vitro [40]. Developmental biology may provide important cues that will reveal novel strategies to mature iPSC-CMs.

**Fig. 4:**
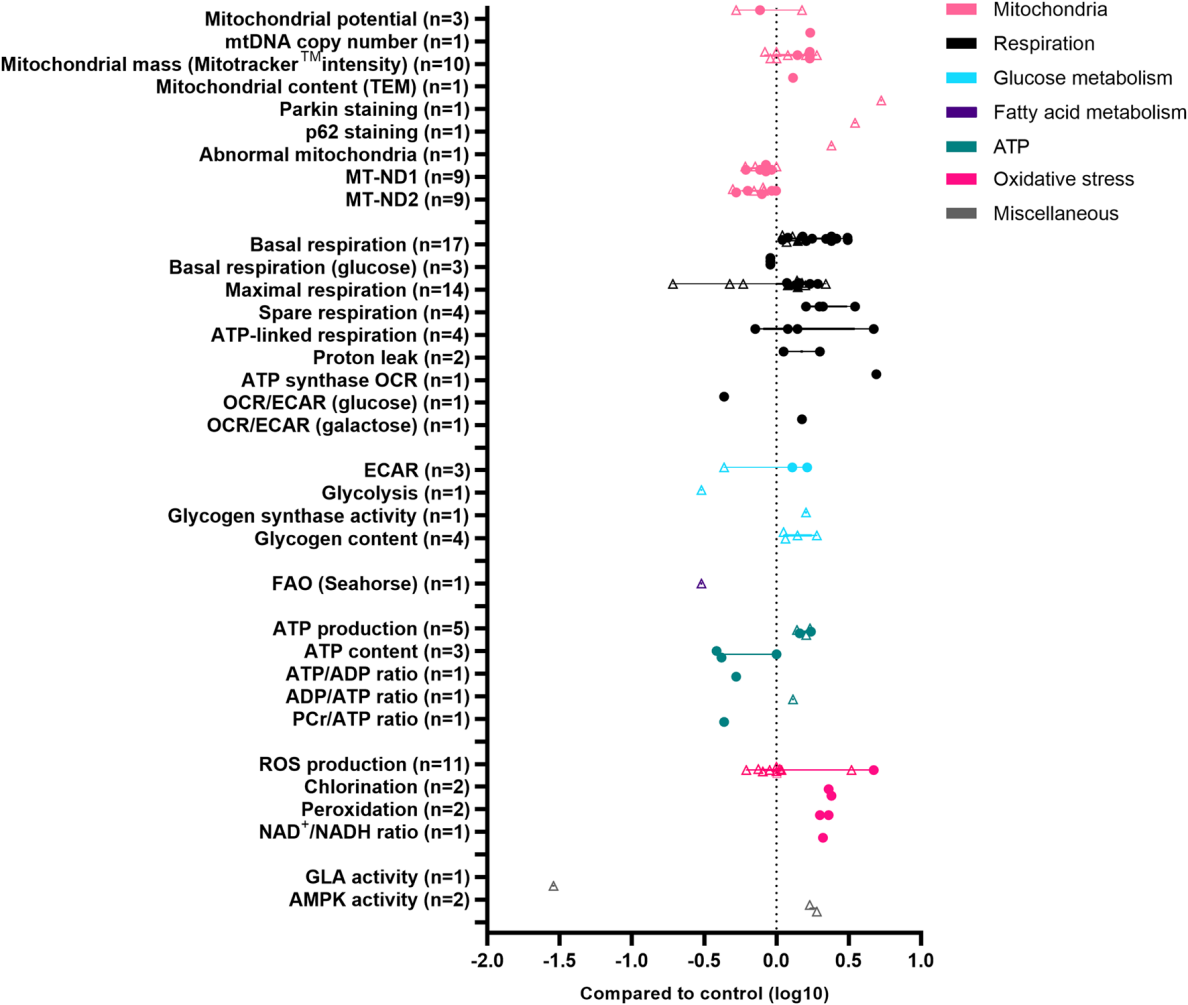
Metabolic characteristics of HCM iPSC-CMs. Overview of changes in metabolic parameters measured in HCM iPSC-CMs and reported in the literature. Each data point represents a log10 fold change compared to control of a single parameter reported in a publication. The quantitative data were obtained from the text, figures or supplemental data of each publication. When the exact value could not be obtained from the publication or its supplements, the quantitative data were retrieved from direct communication with the corresponding author of the respective publication. When no response was received from the authors, the fold change was scored from the bar graphs in the respective study by two reviewers independently, and the mean of the independent assessments was entered as a data point. Circles represent original data, open triangles represent scored data. *ADP* adenosine diphosphate; *GLA* alpha-galactosidase A; *MT-ND1/2* NADH-ubiquinone oxidoreductase chain 1/2; *PCr* phosphocreatine; *TEM* transmission electron microscope

Currently published studies on HCM iPSC-CMs with sarcomere mutations show several of the typical HCM features, such as increased myofilament Ca^2+^-sensitivity [19] and altered cross-bridge kinetics [66]. For the HCM iPSC-CMs with sarcomere mutations, the most reported findings included either increased respiration (5 out of 7 studies) or increased mitochondrial properties (3 out of 7 studies), suggesting a compensatory stage where mitochondrial power increases to meet the elevated energy demand (Fig. 4). Glucose metabolism increased (2 out of 7 studies), but there was limited information available on fatty acid metabolism. Therefore, we could not conclude whether any of the iPSC-CM models displayed the switch from FAO to glycolysis for energy production, which is associated with HCM progression. Cellular hypertrophy was observed in all studies, both in patient-specific iPSC-CMs and in healthy iPSC-CMs where a heterozygous pathogenic mutation was introduced. Increase in cellular size or area varied between studies: 12–51% [59], ~ 33% [12], ~ 24–86% in *MYH7*-mutated iPSC-CM lines [84], ~ 24–38% in *TNNT2*-mutated iPSC-CMs [68] and 76–181% in iPSC-CM lines with a *MYBPC3* mutation [69]. In comparison, intraventricular septum thickness in healthy individuals ranges from 8 to 9 mm [42] and may increase to 13–15 mm in patients before being classified as HCM—an increase of 44–88% [25, 56]. Despite the large variation in the increase in cell size and area in iPSC-CMs, hypertrophy is a common finding in iPSC-CM-based models of HCM [19]. However, metabolic remodeling has been shown to precede the onset of cardiac hypertrophy in individuals with heterozygous sarcomere mutations [16, 29, 63]. These studies indicate that hypertrophy occurs secondary to the metabolic dysfunction in mutation carriers. The presence of hypertrophy in freshly generated iPSC-CMs after introducing a heterozygous HCM mutation might complicate the study of early mutation-mediated changes in metabolism that occur before the onset of cellular hypertrophy. Nevertheless, iPSC-CMs provide valuable insight into the cellular metabolic changes that occur in the diseased cardiomyocyte [91] and provide a useful tool to examine, for example, the effects of gene editing and novel therapeutics in a high-throughput setting.

For the iPSC-CMs of metabolic disorders, no metabolic signature could be defined based on the studies compiled in this overview. Each study investigated different diseases, types of mutations and measured differed metabolic characteristics. The findings in iPSC-CMs of metabolic diseases include decreased respiration, decreased membrane potential, decreased FAO and glycolysis, a decrease in ATP concentration and an increase in oxidative stress. These findings indicate an advanced disease stage with multiple signs of energy depletion, which is further exemplified by the inability to restore basal or maximal respiration after correcting the genetic defect [11, 32, 89]. Of note, the metabolic disease iPSC-CMs with the most severe phenotypes and signs of energy depletion were matured by long-term culture until day 60 [11, 32]. The other iPSC-CM studies included in this overview, including the ones on sarcomere mutations, were measured between day 25 and 40 post-differentiation. Some studies employed 3D culture, but did not assess the effect on maturity of the iPSC-CMs or the disease severity. Therefore, we cannot conclude whether the maturation techniques (long-term culture and 3D culture) influenced the presentation of the disease phenotype. Recently, maturing Danon disease iPSC-CMs by maturation medium led to a more pronounced disease phenotype with increased hypertrophy and reduced tension generation [44], indicating the importance of further investigating the role of maturation in disease modeling.

For future iPSC-CM maturation studies we advise to include assays to assess cardiac metabolism, depending on the research question, budget and available expertise. A basal characterization of cardiac metabolism in iPSC-CMs should include information on substrate use, for which Seahorse assays are a relatively easy readout. Supplementing the Seahorse assays with metabolomics provides detailed insight into the metabolic phenotype of the cardiomyocyte and could be further supported by targeted gene expression. Since Seahorse assays measure oxygen consumption, which only partially reflects substrate metabolism, flux analysis with radioactive or stable isotopes is advised when more detailed information on the activity in certain metabolic pathways is required. Substrate use and metabolic flexibility can further be tested by challenging the cardiomyocyte with different substrates, metabolic activators, inhibitors and biochemical cues, such as insulin.

For future studies on HCM in iPSC-CMs, we advise to focus on mitochondrial (dys)function, which has been considered as a primary event in metabolic remodeling [10]. Other metabolic changes such as the switch of substrate metabolism from FAO to glycolysis and the increase in oxidative stress occur in response to the mitochondrial dysfunction. Therefore, we would advise to examine the mitochondrial (dys)function by, for example, Seahorse as a primary objective, by measuring the oxidative capacity to determine the energetic state of HCM iPSC-CMs.

## Conclusions

In conclusion, this overview adds to the consensus in the field of iPSC-CMs that maturation strategies improve the metabolism of iPSC-CMs, but do not yet achieve an adult cardiomyocyte phenotype. In sarcomeric HCM disease modeling, current iPSC-CMs might prove more useful to test the effects of gene editing and novel therapeutics rather than to identify early disease modifiers. Overall, iPSC-CMs clearly demonstrate their potential to study cardiac metabolism in vitro and future studies in maturation and disease modeling will only offer us more insight into cardiomyocyte (patho)physiology.

### Supplementary Information

**Additional file 1: Figure S1**: Seahorse analyses to measure cardiomyocyte metabolism. A: OCR can be used to determine the role of oxygen consumption in cellular respiration. Basal respiration: oxygen consumption of the cell at baseline conditions. ATP-linked respiration (or ATP turnover) is the proportion of basal respiration that is used for ATP production. Proton leak are protons that return to the mitochondrial matrix independent of the ATP synthase, caused by incomplete coupling of substrate oxidation and ADP phosphorylation. Proton leak can be considered as the basal respiration that is not coupled to ATP production, and it can be calculated as the difference between basal and ATP-linked respiration. Maximal respiration is the maximum oxygen consumption by the ETC. The spare respiratory capacity indicates the cardiomyocyte’s ability to respond to increasing energy demand. It can be calculated as the difference between maximal and basal respiration. Non-mitochondrial oxygen consumption are processes outside of the mitochondria that consume oxygen. B: The ETC in the mitochondrial membrane comprising of five transmembrane proteins (Complex I–IV & Complex V (ATP synthase). Oligomycin: inhibits Complex V by decreasing electron flow causing a reduction in OCR. It can be used to isolate ATP production. FCCP: uncoupling agent. Electron flow through the ETC is unlimited allowing maximal oxygen consumption. It can be applied to measure maximal respiration. Rotenone: inhibits Complex I and Antimycin A inhibits Complex III. Together mitochondrial respiration is decreased to a minimum, which allows determination of non-mitochondrial respiration. C: ECAR can be used to isolate the glycolytic processes in the cardiomyocyte and is determined by measuring proton excretion. Glucose injection stimulates the cardiomyocyte to catabolize glucose. The resulting increase in ECAR indicates glycolysis (or glycolytic rate). ECAR before glucose injection is a measure for non-glycolytic acidification, attributed to processes other than glycolysis that excrete protons. Oligomycin inhibits mitochondrial ATP production, reducing energy production through oxidative phosphorylation and increasing energy production through glycolysis to a maximum, providing a measure for the maximal glycolytic capacity. Glycolytic reserve is the ability of the cardiomyocyte to respond to increased energy demand and is the difference between maximal glycolytic capacity and glycolysis. Adding 2-DG, a glucose analog, completely inhibits glycolysis. The resulting decrease in ECAR confirms that the proton excretion is indeed caused by glycolysis in the cardiomyocyte. D: Assay to determine the proportion of cellular respiration linked to FAO. Four conditions are measured: BSA as a control, fatty acids such as palmitate, both with and without the FAO-inhibitor Eto. Basal, maximal and non-mitochondrial oxygen consumption are similar to (A) but then specific for fatty acid oxidation. Figures are based on the figures from the user guides of Agilent Technologies, Santa Clara, California, and the USA. FCCP = Carbonyl cyanide-4 (trifluoromethoxy) phenylhydrazone; Eto = etomoxir. **Figure S2**: Flux analysis to measure metabolites in the cardiomyocyte. Schematic overview of metabolic flux analysis using ^13^C glucose as an example (Long & Antoniewicz, 2019). A: The first step is isotope tracer design to determine the most optimal isotope for further experiments. Then cells are cultured in presence of the isotope after which external rate measurements are performed. Labeled metabolites are measured using mass spectrometry, for example by GC–MS or LC–MS. Results from the labeling experiments are fitted into a metabolic network model, which can then be used to estimate the flux. Statistical analysis is applied to determine how well the data fit into the model. B: Result of the flux analysis, exemplified for glucose that enters glycolysis and the TCA cycle. Each reaction then yields a number for the flux present in that pathway. Flows are reported as mmol/g_DW_/h. GC–MS = Gas chromatography–mass Spectrometry; LC–MS = Liquid chromatography–mass spectrometry; G6P = Glucose 6-phosphate; P5P = Pentose 5-phosphate; F6P = Fructose 6-phosphate; DHAP = Dihydroxyacetone phosphate; FBP = Fructose 1,6-biphosphate; G3P = Glyceraldehyde 3-phosphate; PGA = 3-Phosphoglyceric acid; PEP = Phosphoenolpyruvic acid; Pyr = Pyruvate; AcCoA = Acetyl CoA; Cit = Citrate; AKG = α-Ketoglutarate; SucCoA = Succinyl CoA; Suc = Succinate; Fum = Fumarate; Mal = Malate; OAC = Oxaloacetate. **Table S1**: Metabolic characteristics of matured iPSC-CMs. Overview of all studies on maturation in iPSC-CMs that measure quantitative metabolic properties. The values that are depicted as ‘fold’ increase or decrease, are calculated as ratios between matured and control iPSC-CMs. Values were retrieved from the text of the papers or requested from the authors of the respective studies. When no response was obtained from the authors, the values were estimated from the bar graphs in the figures of the papers by two independent observers. Values were not allowed to vary more than 10% between observers. Values were averaged and the average is depicted by ~ . Values that do not change more than 1.04-fold are depicted by ± . n.s. = non-significant findings. ACAA2 = Acetyl CoA acyltransferase 2; ACACA/B = Acetyl-CoA carboxylase A/B; ACAD = Acyl-CoA dehydrogenase; ACADM = medium-chain acyl-CoA dehydrogenase; ACADVL = Acyl-CoA Dehydrogenase Very Long Chain; ACAT1 = Acetyl-CoA acetyltransferase 1; ACC = Acetyl-CoA carboxylase; ACFs = adult cardiac fibroblasts; ACL(Y) = ATP citrate lyase; ACO = Acyl-CoA oxidase; ACOT = Acyl-Coa thioesterase 1; ACSL1/3/4 = Long-chain acyl-CoA synthetase 1/3/4; ACSS2 = Acyl-CoA synthetase short chain family member 2; AKAP1 = A-kinase anchoring protein 1; Akt = Protein kinase B; ALDH = Aldehyde dehydrogenase; ALDOA/C = Fructose-biphosphate aldolase A/C; AMPK = AMP-activated protein kinase; ATP5a/b = ATP Synthase; ATP5G3 = ATP synthase H + transporting, mitochondrial F0 complex, subunit C3; ATP5J = ATP synthase-coupling factor 6;ATPAF1 = ATP synthase mitochondrial F1 complex assembly factor 1; ATPG = ATP synthase gamma chain; BABP2 = Bile acid-binding protein 2; BECN1 = Beclin I; BPGM = Biphosphoglycerate mutase; CD36 = Cluster of Differentiation 36 – also known as fatty acid translocase CD36 (FAT-CD36); COX (3/5B/10/17) = Cardiac cytochrome C oxidase (subunit 3/5B/10/17); CPT1A/B, CPT2 = Carnitine Palmitoyltransferase 1 alpha/beta, 2; CS = Citrate synthase; CYP27A1/CYP4F12 = Cytochrome P450 Family 27/4 Subfamily A/F Member 1/12; Cyt-C = Cytochrome C;DGAT1 = DiGlyceride Acyltransferase 1/2; DNM1L = Dynamin 1-like; ECH1 = Enoyl-CoA hydratase 1; ECHS1 = Enoyl-CoA hydratase, short chain 1; ECI1 = Enoyl-CoA delta isomerase 1; ENO1/2 = Enolase 1/2; ESCH = Enoyl-CoA hydratase; ESRRA/G = Estrogen related receptor alpha/gamma; ETFDH = Electron transfer flavoprotein dehydrogenase FABP3 = Fatty acid-binding protein 3; FADS2 = Fatty acid desaturase 2; FASN = Fatty acid synthase; FH = Fumarate hydratase; FIS1 = Mitochondrial fission 1 protein; GABPA = GA binding protein transcription factor subunit alpha; GAPDH = Glyceraldehyde-3-phosphate dehydrogenase; GFAT2 = glutamine-fructose-6-phosphate aminotransferase 2; GLUT1/4 = Glucose transporter type 1/4; GP6PD = glucose-6-phosphate dehydrogenase; HADHA/B = Hydroxyacyl-CoA dehydrogenase trifunctional multienzyme complex subunit A/B; HBP = Hexosamine Biosynthesis Pathway; HIF-1 alpha = Hypoxia-inducable factor 1-alpha; HK2 = Hexokinase 2; IDH1/3A = Isocitrate dehydrogenase 1/3A; iPSC-ECs = induced pluripotent stem cell-derived endothelial cells; iPSC-CFs = induced pluripotent stem cell-derived cardiac fibroblasts; LCAD = Long-chain acyl-CoA dehydrogenase; LDHA = Lactate dehydrogenase A; LPL = Lipoprotein lipase; MAP1LC3A = Microtubule Associated Protein 1 Light Chain 3 Alpha; MCAD = Medium-chain acyl-CoA dehydrogenase; MCT4 = monocarboxylate transporter 4; MFF = Mitochondrial Fission Factor; MFN1/2 = Mitofusion 1/2; MLYCD = Malonyl-CoA decarboxylase, mitochondrial; MPC1 = Mitochondrial pyruvate carrier; mtND1/2 = Mitochondrially encoded NADH:ubiquinone oxidoreductase core subunit 1/2; NDUB9 = NAD dehydrogenase (ubiquinone) 1 beta subcomplex subunit 9NDUF = NADH dehydrogenase ubiquinone flavoprotein; NDUFA1/C2/S3 = NADH:Ubiquinone oxidoreductase subunit A1/C2/core subunit S3;NFE2L2 = Nuclear factor (erythroid-derived 2)-like 2; NRF1 = Nuclear respiratory factor 1; OGDHL = Oxyglutarate dehydrogenase; OPA1 = Optic Atrophy 1; OXCT1 (SCOT) = 3-Oxoacid CoA-transferase 1; PPP = Pentose Phosphate Pathway; P(PAR)GC-1A/B = Peroxisome proliferator-activated receptor Gamma Coactivator-1 alpha/beta; PARK2 = Parkin; PDH = pyruvate dehydrogenase; PDK1/4 = Pyruvate Dehydrogenase Kinase 1/4; PDP1 = pyruvate dehydrogenase phosphatase-1; PFKm/l = Phosphofructokinase, muscle/liver; PGAM1/4 = Phosphoglycerate mutase 1/4; PGD = 6-phosphogluconate dehydrogenase; PGK1 = Phosphoglycerate kinase 1; PKM2 = Pyruvate kinase M2; PPARA/D/G = Peroxisome Proliferator-Activated Receptor alpha/delta/gamma; PRKAA2 = Protein kinase AMP-activated catalytic subunit alpha 2;SCD = Stearol-CoA desaturase; SCOT1 = Sunnicyl-CoA:3-ketoacid coenzyme A transferase 1; SCP = Sterol carrier protein; SDH = Succinate dehydrogenase; SDHC = Succinate dehydrogenase complex subunit C; SFs = skin fibroblasts; SLC2A1/A3/A4/A6/A12/A6, SLC24A6, SLC25A1/A20/A29, SLC27A6 = Solute Carrier (Family) (Member); SREBF1 = Sterol regulatory element-binding factor 1; STC1 = Stanniocalcin-1;SUCLG = Succinyl-CoA ligase [GDP-forming]; TBF2M = Mitochondrial transcription factor B2; TFAM = Transcription factor A mitochondrial; THIM = 3-ketoacyl-CoA thiolase; TPI1 = Triosephosphate isomerase; UCP2 = Mitochondrial uncoupling protein 2. *See “Appendix” for additional information. **Table S2**: Metabolic characteristics of HCM iPSC-CMs. Overview of all studies in iPSC-CMs on HCM and metabolic syndromes with a cardiomyopathy phenotype that measure quantitative metabolic properties. Information on the type of iPSC-CMs that are used as control and hypertrophic properties are also included. The values that are depicted as ‘fold’ increase or decrease, are calculated as ratios between HCM and control iPSC-CMs. Values were retrieved from the text of the papers or requested from the authors of the respective studies. When no response was obtained from the authors, the values were estimated from the bar graphs in the figures of the papers by two independent observers. Values were not allowed to vary more than 10% between observers. Values were averaged and the average is depicted by ~ . Values that do not change more than 1.04-fold are depicted by ± . n.s. = non-significant findings. ACTC1 = Actin Alpha Cardiac Muscle 1; BNP = Brain Natriuretic Peptide; CD36 = Cluster of Differentiation 36; CMTs = Cardiac microtissues; CPT1A/B, CPT2 = Carnitine Palmitoyltransferase 1 alpha/beta, 2, ESRRA = Estrogen related receptor alpha; FABP3 = Fatty acid-binding protein 3; F6P = Fructose-6-phosphate; GLA = α-Galactosidase A; GLUT4 = Glucose transporter type 4; GP = glycogen phosphorylase; GYS1 = Glycogen Synthase 1; G1P = Glucose-1-Phosphate; G6P = Glucose-6-Phosphate; hEHTs = Human engineered heart tissues; HNF4 = Hepatocyte Nuclear Factor 4; LAMP-2 = Lysosomal Associated Membrane Protein type-2; MPO = Myeloperoxidase; MT-ATP6P1 = Mitochondrially encoded ATP synthase 6 pseudogene 1; MT-ATP8 = Mitochondrially encoded ATP synthase membrane subunit 8; MT-CO2 = Mitochondrially encoded cytochrome C oxidase II; MT-CYB = Mitochondrially encoded cytochrome B; MT-ND4L/5/6 = Mitochondrially encoded NADH:ubiquinone oxidoreductase core subunit 4L/5/6; MT-RNR1 = Mitochondrially encoded 12 s RRNA; MT-TP = Mitochondrially encoded TRNA-Pro; MYBPC3 = Myosin-binding protein C3; MYH6 = Myosin heavy chain alpha isoform; MYH7 = Myosin heavy chain beta isoform; NFAT = Nuclear factor of activated T cells; NRF1 = Nuclear respiratory factor 1; PDK4 = Pyruvate Dehydrogenase Kinase 4; PFK-1 = Phosphofructokinase 1; PPARGC1A = Peroxisome proliferator-activated receptor Gamma Coactivator-1 alpha; PPARA/G = Peroxisome Proliferator-Activated Receptor alpha/gamma; PRKAG2 = Protein Kinase AMP-Activated Non-Catalytic Subunit Gamma-2; SLC2A1/A4 =  = Solute Carrier (Family) (Member); TAZ = Tafazzin; TFAM = Transcription factor A mitochondrial; TNNT2 = Troponin T2, Cardiac Type; 1,3BPG = 1,3-bisphosphoglycerate; 3PG = 3-phosphoglycerate

## Data Availability

Not applicable.

## Appendix: Excluded papers and values

After inclusion of the selected studies, we extracted the data and calculated the ratios between matured and control induced pluripotent stem cell-derived cardiomyocytes (iPSC-CMs), reported as fold change. Extreme values were subjected to additional analyses to determine if these values could be included into this review.

Mills et al. [58] measured respiratory activity and fatty acid oxidation (FAO) activity and reported high fold changes for the spare respiratory activity (60x) and fatty acid oxidation oxygen consumption rate (OCR) (17x). After examining the Seahorse data traces, we noticed a high basal (leak) respiration in the control iPSC-CMs compared to the matured iPSC-CMs. A high leak respiration indicates protons migrating into the matrix without producing adenosine triphosphate (ATP), thereby suggesting inefficient coupling between substrate oxidation and ATP synthesis. A high leak respiration leads to a high baseline value, which inherently skews all other measurements (e.g., maximal respiration and spare respiratory capacity), leading to very small values in control iPSC-CMs. Comparing control and mature iPSC-CMs then results in extremely high fold changes. Since the control iPSC-CMs were used in all Seahorse experiments, we excluded these measurements from our additional analyses [58].

Horikoshi et al. [35] measured glucose metabolism in control and matured iPSC-CMs and reported a high fold increase (82x) for the glycolytic reserve in matured iPSC-CMs. Since not many studies examined glycolytic reserve, we could not mirror the value to other values to identify outliers. Therefore, we examined the Seahorse data traces, where we could not observe any response to glucose injection in the control iPSC-CMs. Since control iPSC-CMs are considered ‘immature,’ one would expect an increase in glycolysis as they are known to predominantly use glycolysis for ATP production. The lack of a response in the control iPSC-CM lines and an increased response in the matured iPSC-CMs might therefore explain the large fold change. Since the control iPSC-CM lines were used for all the glucose metabolism measurements (glycolysis, glycolytic capacity), we excluded these values from our additional analyses [35].

Funakoshi et al. [22] measured the respiratory capacity in iPSC-CMs after addition of palmitate to determine the proportion of FAO responsible for respiration. They reported high fold increases for basal and spare respiratory activity (12.5 and 86.7, respectively). The user guide of Seahorse XF Substrate Oxidation Stress Test dictates the use of bovine serum albumin (BSA) as a control, e.g., palmitate as a substrate and FAO-inhibitor etomoxir with both BSA and palmitate to determine the proportion of respiration driven by FAO. However, Funakoshi et al. did not report the BSA + etomoxir measurements. Palmitate + etomoxir completely abolishes respiration, while BSA also shows respiratory activity. Without the BSA + etomoxir condition, the reported respiratory activity cannot solely be attributed to FAO in these iPSC-CMs. Therefore, we have decided to exclude these values from our additional analyses [22].

## Abbreviations

AKT: Protein kinase B
AMPK: 5’AMP-activated protein kinase
ATP: Adenosine triphosphate
Ca^2+^: Calcium
CACT: Carnitine-acylcarnitine translocase
cAMP: Cyclic adenosine monophosphate
CPT1/2: Carnitine palmitoyltransferase 1/2
cTnC: Troponin C
ECAR: Extracellular acidification rate
ERK: Extracellular signal-regulated kinase
ETC: Electron transport chain
FA(s): Fatty acid(s)
FAO: Fatty acid oxidation
FAT/CD36: Fatty acid translocase/cluster of differentiation 36
HCM: Hypertrophic cardiomyopathy
HIF-1α: Hypoxia-inducible factor 1 alpha
iPSC-CMs: Induced pluripotent stem cell-derived cardiomyocytes
LAMP-2: Lysosomal-associated membrane protein-2
MAPK: Mitogen-activated protein kinase
MMP: Mitochondrial membrane potential
MPO: Myeloperoxidase
mTOR: Mechanistic target of rapamycin
NCX: Sodium–calcium exchanger
OCR: Oxygen consumption rate
PI3K: Phosphoinositide 3-kinase
PPAR: Peroxisome proliferator-activated receptor
RNA seq: RNA sequencing
ROS: Reactive oxygen species
RT-qPCR: Quantitative reverse-transcription polymerase chain reaction
RyR: Ryanodine receptor
SR: Sarcoplasmic reticulum
SERCA: Sarco-endoplasmic reticulum Ca^2+^ ATPase
TCA cycle: Tricarboxylic acid cycle
TMRE/M: Tetramethylrhodamine (m)ethyl ester
