# Supplementary material for: Characterization of cardiac metabolism in iPSC-derived cardiomyocytes: lessons from maturation and disease modeling
Source: Stem Cell Res Ther. 2022 Jul 23;13:332. doi: 10.1186/s13287-022-03021-9 (PMC9308297; doi:10.1186/s13287-022-03021-9)
Supplement: Supplementary file 1 — Additional file 1: Figure S1: Seahorse analyses to measure cardiomyocyte metabolism. A: OCR can be used to determine the role of oxygen consumption in cellular respiration. Basal respiration: oxygen consumption of the cell at baseline conditions. ATP-linked respiration (or ATP turnover) is the proportion of basal respiration that is used for ATP production. Proton leak are protons that return to the mitochondrial matrix independent of the ATP synthase, caused by incomplete coupling of substrate oxidation and ADP phosphorylation. Proton leak can be considered as the basal respiration that is not coupled to ATP production, and it can be calculated as the difference between basal and ATP-linked respiration. Maximal respiration is the maximum oxygen consumption by the ETC. The spare respiratory capacity indicates the cardiomyocyte’s ability to respond to increasing energy demand. It can be calculated as the difference between maximal and basal respiration. Non-mitochondrial oxygen consumption are processes outside of the mitochondria that consume oxygen. B: The ETC in the mitochondrial membrane comprising of five transmembrane proteins (Complex I–IV & Complex V (ATP synthase). Oligomycin: inhibits Complex V by decreasing electron flow causing a reduction in OCR. It can be used to isolate ATP production. FCCP: uncoupling agent. Electron flow through the ETC is unlimited allowing maximal oxygen consumption. It can be applied to measure maximal respiration. Rotenone: inhibits Complex I and Antimycin A inhibits Complex III. Together mitochondrial respiration is decreased to a minimum, which allows determination of non-mitochondrial respiration. C: ECAR can be used to isolate the glycolytic processes in the cardiomyocyte and is determined by measuring proton excretion. Glucose injection stimulates the cardiomyocyte to catabolize glucose. The resulting increase in ECAR indicates glycolysis (or glycolytic rate). ECAR before glucose injection is a measure for non-glycolytic aci [file 13287_2022_3021_MOESM1_ESM.pdf]

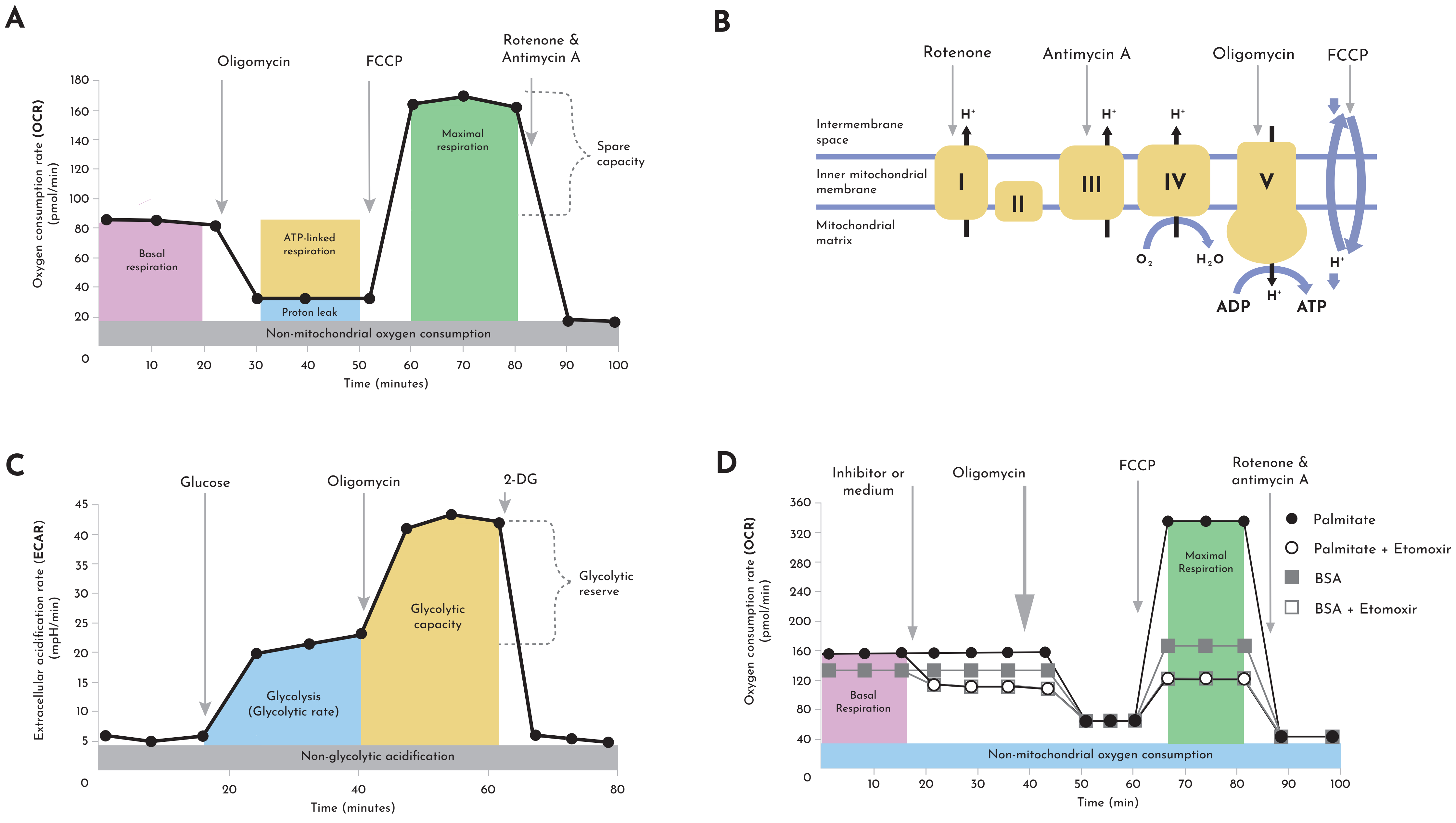

Figure S1: Seahorse analyses to measure cardiomyocyte metabolism

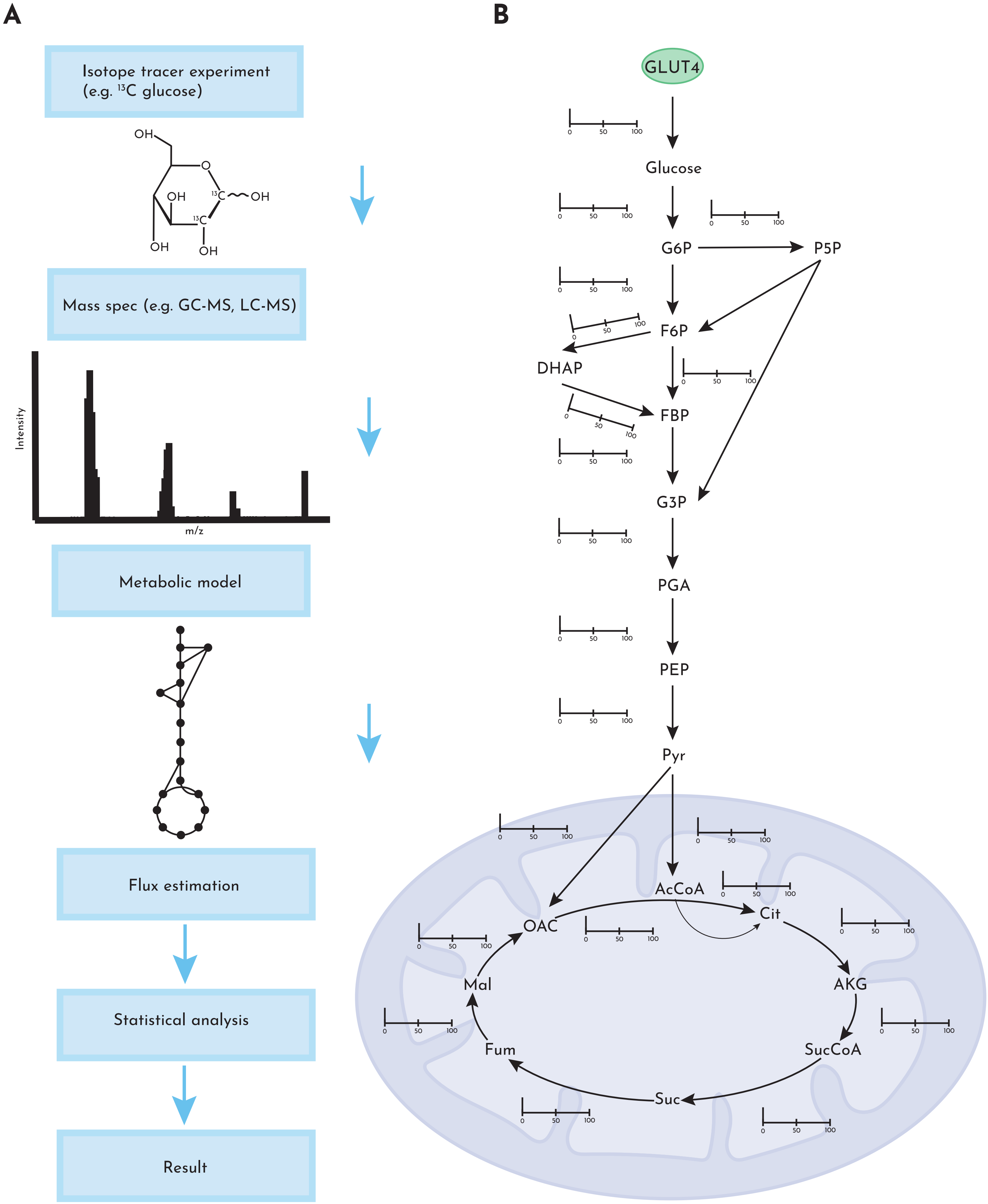

Figure S2: Flux analysis to measure metabolites in the cardiomyocyte

Table S1: Metabolic characteristics of matured iPSC-CMs

Author	Maturation protocol	Gene expression	Protein levels	Mitochondria	Respiratory capacity	Glycolysis	Glucose metabolism	Fatty acid metabolism	ATP	Other cycles and pathways
Bekhite et al. (2020)	Prolonged culture for 28 days, compared to 9 days	Glucose metabolism ↑ (*PDK4*)<br><br>Oxidative metabolism ↑ (*DGAT1*)<br><br>FA metabolism ↑(*CD36*, *PPARA*, *CPT1B*)<br><br>Mitochondrial biogenesis (*PPARGC1A*), fission (*MFF*) ↓ and fusion (*MFN2*, *OPA1*) ↑	Proteins involved in FA uptake and oxidation (PPARα, CPT-1B) and mitochondrial biogenesis (PGC-1α) ↑	Mitochondrial size, mitochondrial network ↑<br><br>Amount of tubularly shaped mitochondria ↑<br><br>Maximum length-to-width ratio of mitochondria ↑<br><br>Developed cristae ↑<br><br>Mitotracker™ intensity ↑ (~3.3-fold)	Basal respiration ↑ (~1.9-fold)<br><br>Maximal respiration ↑ (~2-fold)<br><br>Spare respiratory capacity ↑ (~2.2-fold)<br><br>Proton leak ↑ (~2.2-fold) (n.s)				ATP production ↑ (~2.5-fold)	
	Lactate metabolic purification of 21-day-old iPSC-CMs for 5 days				Basal respiration ↑ (2.8-fold)<br><br>Maximal respiration ↑ (2.1-fold)<br><br>Spare respiration ↑ (1.8-fold)<br><br>Proton leak ↑ (~1.2-fold) (n.s.)	ECAR ↑ (1.8-fold)			ATP production ↑ (2.7-fold)	

Table S1: Metabolic characteristics of matured iPSC-CMs

Author	Maturation protocol	Gene expression	Protein levels	Mitochondria	Respiratory capacity	Glycolysis	Glucose metabolism	Fatty acid metabolism	ATP	Other cycles and pathways
Bhute et al. (2017)	2D<br>Extended culture for 90 days, compared to 30 days				Maximal respiration ↑ (~2-fold)<br><br>Basal OCR upon CPT-1 inhibition ↓			β-oxidation of branched and very long chain fatty acids ↑		Glycerol phosphate shuttle, nicotinate and nicotinamide metabolism, biotin and taurine metabolism, lysine degradation, pantothenate and CoA biosynthesis, β-alanine and sphingolipid metabolism ↑<br><br>Correlation of metabolic pathways involving amino acids, pyruvate and glucose metabolism ↓<br><br>Connectivity of phospholipid metabolism-related metabolites ↑

Table S1: Metabolic characteristics of matured iPSC-CMs

Author	Maturation protocol	Gene expression	Protein levels	Mitochondria	Respiratory capacity	Glycolysis	Glucose metabolism	Fatty acid metabolism	ATP	Other cycles and pathways
Correia et al. (2017)	2D<br>Metabolic substrate manipulation<br>(1) GFAM d20 (+15)<br>Compared to<br>(2) GLCM d20	FA metabolism ↑ (*CD36, CPT1B, SLC25A20, HADHA/B*)<br><br>Oxidative phosphorylation ↑ (*ATP5, COX family, NDUF family*)<br><br>TCA cycle ↑ (*ACO2, IDH3A, SUCLG1, FH, OGDHL)*		Mitochondrial membrane potential ↑ (~1.4-fold)	Basal OCR ↑ (~1.6-fold)	ECAR ↓ (~4.1-fold)	PDH activity ↑ (12.9-fold)<br><br>Lactate secretion ↓ (100%)	Contribution of FAs to TCA cycle ↑ (~1.9-fold for (1) compared to day 0)	ATP production ↑ (2.7-fold)	

Table S1: Metabolic characteristics of matured iPSC-CMs

Author	Maturation protocol	Gene expression	Protein levels	Mitochondria	Respiratory capacity	Glycolysis	Glucose metabolism	Fatty acid metabolism	ATP	Other cycles and pathways
Emanuelli et al. (2022)	2D<br>Prolonged culture, from 6 to 12 weeks		Glucose transporters GLUT1 and GLUT4 ±<br><br>Lactate transporter MCT4 ↑<br><br>Glycolytic enzymes PFKM, HK1 ↑, HK2 ↓<br><br>TCA flux-related enzymes PDH, PDK1, PDK4, PDP1 ↑<br><br>HBP enzyme GFAT2 ↓<br><br>PPP enzymes G6PD, PGD ↓<br><br>Enzymes involved in FA metabolism (PPARα, CD36, ACADM, HADH, ACC2) ↑<br><br>ETC proteins ±	mtDNA/nDNA ↑ (~1.4-fold)<br><br>Mitochondrial membrane potential ↑ (~1.2-fold)<br><br>Mitochondrial calcium ↑ (~1.2-fold)<br><br>Mitochondrial footprint (density) ↑ (~1.1-fold) (n.s.)<br><br>Mitochondrial branch length ↑ (~1.4-fold)<br><br>Number of branches ↑ (~1.3-fold)	OCR/ECAR ratio ↑ (~1.5-fold)<br><br>Respiration reserve (pyruvate) ↑ (~1.8-fold)<br><br>Respiration reserve (glucose) ↑ (~2.2-fold)<br><br>Respiration reserve (lactate) ↑ (~3.2-fold)<br><br>Respiration reserve (palmitate) ↑ (~2.5-fold)	Glycolysis ↑ (~1.1-fold) (n.s.)<br><br>Glycolytic reserve ↓ (~1.1-fold) (n.s.)	Anaerobic glycolytic flux ± (n.s.)<br><br>PPP flux ↓ (~1.3-fold) (n.s.)<br><br>Glucose oxidation ↓ (~1.9-fold)	FAO (Seahorse) ↑ (~1.8-fold)		Glutamine oxidation ±<br><br>Principal component analysis: proximal glycolytic intermediates ↓, pyruvate and TCA cycle precursors ↑

Table S1: Metabolic characteristics of matured iPSC-CMs

Author	Maturation protocol	Gene expression	Protein levels	Mitochondria	Respiratory capacity	Glycolysis	Glucose metabolism	Fatty acid metabolism	ATP	Other cycles and pathways
Funakoshi et al. (2021)	2D<br>Addition of palmitate, a PPARα agonist (P), dexamethasone (D) and T3 (T)	CD36 ↑	Lipid droplets		In response to exogenous palmitate:<br><br>Basal respiration ↑ *<br><br>Maximal respiration ↑*<br><br>Proton leak ↑*				In response to exogenous palmitate:<br><br>ATP production ↑*	
	Culture for 9 days on low glucose and PPDT + 5 days on PPDT (1), PP (2), palmitate (3) or only low glucose (4)				In response to exogenous palmitate:<br><br>Maximal respiration ↑ (for (3) compared to (1)) *<br><br>Spare capacity ↑ (for (3) compared to (1)) *				In response to exogenous palmitate:<br><br>ATP production ↑ (for (3) compared to (1)) *	

Table S1: Metabolic characteristics of matured iPSC-CMs

Author	Maturation protocol	Gene expression	Protein levels	Mitochondria	Respiratory capacity	Glycolysis	Glucose metabolism	Fatty acid metabolism	ATP	Other cycles and pathways
Garbern et al. (2020)	2D<br>Addition of mTOR-inhibitor Torin 1	Mitochondrial biogenesis ↑ (*PPARGC1A*)<br><br>FA metabolism ↑ (*SLC27A6*)<br><br>Glucose metabolism ↓ *(GLUT1)*		Mitochondrial membrane potential ↑ (~1.6-fold)<br><br>Mitotracker™ intensity ↓ (~1.5-fold)<br><br>MtDNA/nDNA ↓ (~1.6-fold)	Maximal respiration ↑ (~1.5-fold)<br><br>Spare respiratory capacity ↑ (~3.5-fold)<br><br>Non-mitochondrial respiration ↓ (~1.1-fold) (n.s.)	Glycolysis ↓ (1.1-fold) (n.s.)<br><br>Non-glycolytic acidification ↓ (~1.7-fold)<br><br>Glycolytic capacity ±<br><br>Glycolytic reserve ±				
Horikoshi et al. (2019)	2D<br>7-day lactate purification and subsequent fatty acid supplementation, no glucose, for 3-7 days	FA metabolism ↑ *(PPARA)*		Number of mitochondria associated with myofibrils ↑	Basal respiration ↑ (2.5-fold)<br><br>Maximal respiration ↑ (2.7-fold)<br><br>Spare respiratory capacity ↑ (2.8-fold)<br><br>In response to exogenous palmitate:<br><br>Basal respiration ↑ (4.4-fold)<br><br>Maximal respiration ↑ (2.5-fold)	Glucose-induced glycolysis ↑*<br><br>Glycolytic capacity ↑*<br><br>Glycolytic reserve ↑*			ATP production ↑ (3.6-fold)	

Table S1: Metabolic characteristics of matured iPSC-CMs

Author	Maturation protocol	Gene expression	Protein levels	Mitochondria	Respiratory capacity	Glycolysis	Glucose metabolism	Fatty acid metabolism	ATP	Other cycles and pathways
Hu et al. (2018)	2D<br>Fatty acid supplementation, no glucose for 7 days, compared to culture in glucose media	Lactate dehydrogenase (*LDHA*) ↓	HIF1α ↓	Mitotracker™ intensity ↑ (1.5-fold)	Basal respiration ↑ (1.8-fold)<br><br>Spare respiratory capacity ↑		Hexokinase activity ↓ (5.1-fold)<br><br>Lactate levels ↓ (4.1-fold)<br><br>Nuclear-to-cytoplasmic HIF1α protein ratio ↓		ATP per cell ↑ (2-fold)	
	(1) HIF1α inhibition, (2) LDHA inhibition or (3), combi-nation 1 and 2<br><br>Cultured for 4 days in glucose media (A), glucose + fatty acids media (B), or fatty acid media (C)			mtDNA/gDNA ↑ (3.9-fold for (3) and (B) compared to (B) alone) (no statistics)<br><br>Mitochondria number per area ↑ (2-fold for (1) and (B) compared to (B) alone) (no statistics)	Basal respiration ↑ (3.1-fold for (2) and (B), compared to (B) alone) (no statistics)<br><br>Spare respiratory capacity ↑ (for (3) and (B) compared to (B) alone)		Hexokinase activity ↓ (3.6-fold for (1) and (A) compared to (A) alone)<br><br>Lactate levels ↓ (2.4-fold for (2) and (B) compared to (B) alone)		ATP per cell ↑ (2-fold for (3) and (A) and (B) compared to (A) and (B) alone respectively)	

Table S1: Metabolic characteristics of matured iPSC-CMs

Author	Maturation protocol	Gene expression	Protein levels	Mitochondria	Respiratory capacity	Glycolysis	Glucose metabolism	Fatty acid metabolism	ATP	Other cycles and pathways
Miki et al. (2021)	2D + supplementation of Estrogen-related receptor gamma agonist (T112) (1) + S-phase kinase-associated protein 2 inhibitor (T623) (2) or both (3)	Glucose metabolism genes ↓ (*TPI1, ENO2, PGK1, PGAM1, SLC2A12* etc.)<br><br>FA metabolism-related genes ↑ (*ACACB, SLC25A29, ACLY, ACSL4, ACSS2* etc.)<br><br>Oxidative phosphorylation-related genes ↑ (*NDUF family, COX5B, IDH1* etc.)<br><br>Mitochondrial remodeling ↑ (*TFAM)*		Mitochondrial staining ↑ (1.4-fold for (1)) (no statistics)	Basal respiration ↑ (2.4-fold for (3))<br><br>Maximal respiration ↑ (2-fold for (3) in one iPSC-CM line and 1.5-fold for (1) in another iPSC-CM line)				ATP production ↑ (2.5-fold for (3))	

Table S1: Metabolic characteristics of matured iPSC-CMs

Author	Maturation protocol	Gene expression	Protein levels	Mitochondria	Respiratory capacity	Glycolysis	Glucose metabolism	Fatty acid metabolism	ATP	Other cycles and pathways
Ramachandra et al. (2018)	2D<br>Fatty acid supplementation for 30 days	Mitochondrial remodeling ↑ (*OPA1, MFN1, MFN2, DNM1L, FIS1, NRF1, NFE2L2, TFAM, TBF2M*)<br><br>Mitochondrial turnover ↑ (*PARK2, BECN1, MAP1LC3A*)<br><br>Mitochondrial biogenesis ↑ (*PPARGC1A, ESRRA*)<br><br>FA metabolism↑ (*PPARA, CPT1B, MLYCD*) and AMPK subunit (*PRKAA2*)		Mitochondrial membrane potential ↑ (~1.1-fold) (n.s.)<br><br>Mitochondrial filamentous networks ↑<br><br>Developed cristae ↑<br><br>Mitochondrial size and elongation ↑<br><br>Number of mitochondria located next to the sarcoplasm ↑	Basal respiration ±<br><br>Maximal respiration ↑ (2-fold)<br><br>Spare respiration (5-fold) ↑<br><br>Non-mitochondrial respiration ↑ (~1.1-fold) (n.s.)<br><br>OCR:ECAR ratio ↑			Long-chain acylcarnitine s ↑ (e.g. C16, C18:1),	ATP concentration ↑ (2.3-fold)	TCA cycle intermediates ↑ (α-ketoglutarate, citrate, succinate, fumarate, malate)
	2D<br>30 day extended culture, compared to day 14 in culture		Mitochondrial (OPA1, MFN1, MFN2) and FA metabolism- and complex I-related proteins ↑<br><br>Mitochondrial fission protein FIS1 ↓	Mitochondrial membrane potential ↑<br><br>Mitochondrial filamentous networks ↑<br><br>Developed cristae ↑<br><br>Mitochondrial size and elongation ↑			Hexokinase activity ↓ (1.8-fold)	L-carnitine levels ↑ (3.7-fold)	ATP concentration ↑ (1.7-fold)	

Table S1: Metabolic characteristics of matured iPSC-CMs

Author	Maturation protocol	Gene expression	Protein levels	Mitochondria	Respiratory capacity	Glycolysis	Glucose metabolism	Fatty acid metabolism	ATP	Other cycles and pathways
Yang et al. (2014)	2D<br>Thyroid hormone (T3) supplementation for 7 days			MtDNA/nDNA ±<br><br>Mitochondrial volume ↑ (~1.3-fold) (n.s.)	Basal OCR ↑ (~1.7-fold)<br><br>Maximal OCR ↑ (1.5-fold)<br><br>Respiratory reserve capacity ↑ (~1.5-fold)<br><br>Non-mitochondrial OCR ↑ (~1.6-fold)					
Yang et al. (2019)	2D<br>Fatty acid-albumin complex supplementation for 14 days	FA synthesis ↓ (*SREBF1, FADS2, SCD etc.*)<br><br>FA metabolism ↑ (*CD36, CPT1A, SLC25A20, ACADVL, ECH1, ACAA2 etc.*)<br><br>Glucose metabolism (*PDK4*), electron transfer enzyme (*ETFDH*) ↑<br><br>Glucose transporter (*SLC2A4*), hexokinase (*HK2*) ↓	Phospho- AMPK Thr^{172}, phospho-ERK, phospho-p38 MAPK and phospho-ACC ↑; phospho-Akt ↓		Maximal respiratory capacity ↑ (38%)					

Table S1: Metabolic characteristics of matured iPSC-CMs

Author	Maturation protocol	Gene expression	Protein levels	Mitochondria	Respiratory capacity	Glycolysis	Glucose metabolism	Fatty acid metabolism	ATP	Other cycles and pathways
Ye et al. (2021)	2D<br>AMPK activation during differentiation	FA metabolism ↑ (*CPT1A, CPT1B, FABP3, FAT-CD36, SLC27A6, SLC25A20, LCAD, MCAD*)<br><br>Oxidative phosphorylation ↑ (*COX5b, ATP5a, Cyt-C*)	AMPK, phopho-AMPK, PGC-1α ↑<br><br>FA metabolism-related proteins ↑ (CPT-1α, CPT-1β, SLC24a6)<br><br>Oxidative phosphorylation ↑ (COX5B, COXIV, Cyt-C, CS, MPC1)	Number of mitochondria ↑<br><br>Developed cristae ↑<br><br>mtDNA/nDNA ↑ (~3.8-fold for *mt-ND1* and ~4-fold for *mt-ND2*)<br><br>Mitochondrial membrane potential ↑ (~2-fold)	Basal respiration ↑ (~1.5-fold)<br><br>Maximal respiration ↑ (~1.8-fold)<br><br>Proton leak ↓ (~1.3-fold)<br><br>Non-mitochondrial respiration ↑ (~1.3-fold)		Hexokinase activity ↓ (~2.8-fold)<br><br>Lactate concentration ↓ (~1.3-fold)		ATP production ↑ (~1.4-fold)<br><br>ATP levels ↑ (~1.2-fold)	
Yoshida et al. (2018)	2D<br>Co-culture with non-CMs:<br>(1) CMs<br>(2) CMs + hMSCs<br>(3) CMs + SF<br>(4) CMs + SF 25%<br>(5) CMs + SF 50%, all compared to (1)	Mitochondria-associated ↑ (*NADH, COX3* (highest for (1))<br><br>ROS-protection ↑ ((*STC1*), *for* (3))	STC-1 ↑ (for (3))		Basal respiration ↑ (1.9-fold; (highest for (5))<br><br>Spare respiratory capacity ↑ (2.1-fold; highest for (5))<br><br>Metabolic potential ↑ (1.1-fold; highest for (5))	ECAR ±			ATP production ↑ (1.2-fold; highest for (5))	Ratio in ROS level ↓ (4.6-fold; lowest for (3))

Table S1: Metabolic characteristics of matured iPSC-CMs

Author	Maturation protocol	Gene expression	Protein levels	Mitochondria	Respiratory capacity	Glycolysis	Glucose metabolism	Fatty acid metabolism	ATP	Other cycles and pathways
Feyen et al. (2020)	2D<br>Culture in maturation media for 21-35 days	Mitochondrial biogenesis ↑ (*PPARGC1A*)<br><br>FA metabolism ↑ (*PPARA, PPARD, ACADVL, HADHB, ACSL1*)<br><br>Oxidative metabolism ↑ (*LPL, ACAT1, DGAT1*)		Amount of perinuclear mitochondria ↓<br><br>Amount of perisarcomeric mitochondria ↑<br><br>Tom20 staining intensity ↑ (1.7-fold)	Basal respiration ↑ (1.5-fold) (n.s.)<br><br>Maximal respiration ↑ (1.4-fold)<br><br>Spare respiration ↑ (1.4-fold)	Non-glycolytic acidification ↑ (1.5-fold)<br><br>Glycolytic capacity ↑ (1.7-fold)<br><br>Glycolysis ↑ (2-fold) (n.s.)		Fatty acid uptake ↑ (1.2-fold) (no statistics)	ATP production ↑ (2.9-fold)	
	3D<br>Culture in maturation media for 29-42 days			Development of cristae ↑<br><br>mtDNA/gDNA ↑ (~1.4-fold for both *mt-ND1* and *mt-ND2*)			Ratio lactate production/glucose consumption ↓ (~3-fold) (no statistics)			

Table S1: Metabolic characteristics of matured iPSC-CMs

Author	Maturation protocol	Gene expression	Protein levels	Mitochondria	Respiratory capacity	Glycolysis	Glucose metabolism	Fatty acid metabolism	ATP	Other cycles and pathways
Lopez et al. (2021)	2D Supplementation by (1) oleate (OA) or (2) OA + PPARα agonist WY-14643 for 1 week	Glucose metabolism *SLC2A4* ↓, *PFKM*↓, (lowest for (2)), *PDK4* ↑, highest for (1))<br><br>FA metabolism *(CPT1B* ↑ (highest for (2) after 1 week), *ECI1* ↓ (lowest for (2)))			Basal OCR with pyruvate + malate as substrate ↑ (~3.9-fold; highest for (2))<br><br>Basal OCR with oleate as substrate ↑ (~3.5-fold; highest for (2)) (n.s.)<br><br>Maximal OCR with pyruvate + malate as substrate ↑ (~2-fold; highest for (2)) (n.s.)<br><br>Maximal OCR with oleate as substrate ↑ (~2.6-fold; highest for (2)) (n.s.)	Glycolysis ↓ (~1.1-fold; lowest for (1))		Palmitate oxidation ↑ (~5.6-fold; highest for (2))<br><br>Oleate oxidation ↑ (~1.4-fold; highest for (2))		

Table S1: Metabolic characteristics of matured iPSC-CMs

Author	Maturation protocol	Gene expression	Protein levels	Mitochondria	Respiratory capacity	Glycolysis	Glucose metabolism	Fatty acid metabolism	ATP	Other cycles and pathways
Lopez et al. (2021) *cont.*	3D Supplementation by (1) OA or (2) OA + WY-14643, compared to 2D monolayer	Mitochondrial biogenesis ↑ (*PPARGC1A* for (2))<br><br>FA metabolism ↑ (*PPARA* for (1))<br><br>Glucose metabolism ↑ (*PDK4* for (1))				Glycolysis ↓ (~1.3-fold)<br><br>Glucose oxidation ↑		Palmitate oxidation ↑ (~5.4-fold; highest for (2))<br><br>Oleate oxidation ↑ (~5-fold; highest for (2))		

Table S1: Metabolic characteristics of matured iPSC-CMs

Author	Maturation protocol	Gene expression	Protein levels	Mitochondria	Respiratory capacity	Glycolysis	Glucose metabolism	Fatty acid metabolism	ATP	Other cycles and pathways
Correia et al. (2018)	3D<br>Prolonged culture for 35 days, compared to 15 days	Glucose metabolism ↓ (*SLC2A1, SLC2A3, SLC2A6, LDHA, ALDOA, HK2, PKM2, GAPDH, PGK1, ENO2, PGAM4, PFKL, ENO1, TPI1, ALDOC, PGAM1*)<br><br>Oxidative phosphorylation ↑ (*NDUFA1, SDHC, COX17, COX5B, COX10, ATP5J, ATPAF1, ATP5G3*)<br><br>Mitochondrial uncoupling protein ↓ (*UCP2*)<br><br>Mitochondrial biogenesis ↑ (*ESRRG*)<br><br>Lipid biosynthesis ↓ (*SLC25A1, SREBF1, FASN, ACACA, ACLY, ACSL4, ACSL3, SCD, MLYCD* ↑)<br><br>FA oxidation and transport ↑ (*CD36, CPT1B*)				Glycolysis ↓ (~1.4-fold)<br><br>Glycolytic flux ↓<br><br>Glucose uptake ↓ (30 %)	Lactate production ↓<br>(35 %)<br><br>Pentose phosphate pathway ↓ (1.8-fold)	Fatty acid synthesis ↓	ATP production ↑ (~1.6-fold)<br><br>ATP production from anaerobic glycolysis ↓ (~1.5-fold) (no statistics)<br><br>ATP production from glucose oxidation ↑ (~2-fold) (no statistics)	Carbon metabolism, amino acid synthesis ↓<br><br>Flux of pyruvate to mitochondria ↑ (~4.3-fold)<br><br>TCA cycle ↑ (~1.9-fold)<br><br>Amino acid catabolism ↑

Table S1: Metabolic characteristics of matured iPSC-CMs

Author	Maturation protocol	Gene expression	Protein levels	Mitochondria	Respiratory capacity	Glycolysis	Glucose metabolism	Fatty acid metabolism	ATP	Other cycles and pathways
Correia et al. (2018) *cont.*		Oxidative metabolism ↑ (*LPL)*								
Gentillon et al. (2019)	3D (cardiac spheres)<br>HIF-1α inhibition + PPARα agonist WY-14643<br>+T3, IGF-1 and dexamethasone	FA metabolism ↑ (*ACADVL, CPT1B, BABP2, CD36, PPARA, PGC1A*)<br><br>NADH:ubiquinone Oxidoreductase ↑ (*NDUFC2, NDUFS3)*<br><br>Cytochrome P450 family ↑ (*CYP27A1, CYP4F12)*<br><br>Glucose metabolism↑ *(PDK4, SLC2A4)*<br><br>Mitochondrial uncoupling protein *(UCP3)* ↑<br><br>FA synthase (*FASN*) ↑<br><br>Mitochondrial biogenesis *(ESRRA)* ↑		Mitotracker™ intensity ↑ (1.1-fold)<br><br>Mitochondrial dispersion throughout the cell ↑ (2.5-fold) (no statistics)<br><br>Amount of perinuclear mitochondria ↓ (1.9-fold) (no statistics)	Basal respiration ↑ (2-fold)<br><br>Maximal respiration ↑ (2-fold)<br><br>Reserve capacity ↑ (2-fold)<br><br>Non-mitochondrial respiration ↑ (1.7-fold)<br><br>Proton leak ↑ (2.4-fold)			FAO (Seahorse) ↑ (5-fold)<br><br>Oxidation non-FA substrates ↑ (3-fold)	ATP production ↑ (2-fold)	

Table S1: Metabolic characteristics of matured iPSC-CMs

Author	Maturation protocol	Gene expression	Protein levels	Mitochondria	Respiratory capacity	Glycolysis	Glucose metabolism	Fatty acid metabolism	ATP	Other cycles and pathways
Giacomelli et al. (2020)	3D culture for 21 days with non-CMs:<br><br>(1) iPSC-CMs+ iPSC-ECs<br>(2) = (1) + ACFs<br>(3) = (1)+ SFs<br>(4)=(1) + iPSC-CFs<br>(5) iPSC-CMs+ iPSC-CFs<br><br>Values depicted are compared to (1)	FA metabolism ↑ (*CPT1A, CPT1B, FABP3, ACADM, ACADVL, ECH1* etc.)<br><br>FA biosynthesis ↓ (*FASN, ACC, ACL*)<br><br>Glucose metabolism ↓ (*SLC2A1, HK1, HK2, PFKL, ALDOA, GAPDH, PGK1, ENO1, ENO2*)		Developed cristae ↑ (for (4))<br><br>Mitochondrial size and elongation ↑ (for (4))	Basal consumption ↑ (2.8-fold; highest for (5))<br><br>Maximal respiration ↑ (4.6-fold; highest for (5))<br><br>Spare respiratory capacity ↑ (6.8-fold; highest for (4))<br><br>Proton leak ↑ (6.2-fold; highest for (2)	Basal glycolysis ↑ (1.6-fold; highest for (2))<br><br>Glycolytic capacity ↑ (2.2-fold; highest for (2))<br><br>Glycolytic reserve ↑ (2.6-fold; highest for (2))	Lactate release ↓ (lowest for (2) and (4))<br><br>Glutamine uptake ↑ (highest for (2) and (4))		ATP production ↑ (2.6-fold; highest for (5))<br><br>Intracellular ATP concentration ↑ (highest for (5))	
Li et al. (2020)	3D<br>Self-organized tissue rings with re-entrant waves				Maximal respiration capacity ↑ (~2-fold)					

Table S1: Metabolic characteristics of matured iPSC-CMs

Author	Maturation protocol	Gene expression	Protein levels	Mitochondria	Respiratory capacity	Glycolysis	Glucose metabolism	Fatty acid metabolism	ATP	Other cycles and pathways
Mills et al. (2017)	3D<br>Fatty acid supplementation for 11 days	14 glycolysis genes ↑ (*BPGM, GAPDH, HK2, TPI, ALDOA, ALDOC, LDHA, PGK1 etc.)*	10 glycolysis proteins ↑ (BPGM, GAPDH, HK2, TPI, ALDOA, ALDOC, LDHA, PGK1, etc.)<br><br>7 fatty acid metabolism-related proteins ↑ (ACADM, CPT2, CPT1B, ETFDH etc.)	Mitochondrial development ↑<br><br>mtDNA ↑ (~2.6-fold)	Maximal OCR ↑*<br><br>Reserve OCR ↑*<br><br>OCR with FAs ↑*		Hexosamine, pentose-phosphate, and glycogen pathways ↑			
Ronaldson-Bouchard et al. (2018)	3D, EHT<br>Pacing of early- and late-stage iPSC-CMs (intensity training) with fibroblasts for 28 days	PKA-regulator *AKAP1* ↓<br><br>Mitochondrial biogenesis ↓ *(TFAM, PPARGC1A)*		Mitochondrial density ↑ (3-fold)<br><br>Mitochondrial development and elongation ↑	Maximal respiration ↑<br><br>Respiratory reserve ↑<br><br>Basal OCR ↑	ECAR ↑			ATP turnover ↑	

Table S1: Metabolic characteristics of matured iPSC-CMs

Author	Maturation protocol	Gene expression	Protein levels	Mitochondria	Respiratory capacity	Glycolysis	Glucose metabolism	Fatty acid metabolism	ATP	Other cycles and pathways
Ulmer et al. (2018)	3D<br>Culture for 21-45 days		Abundance of FAO enzymes ↑ (ACOT1, ACOT13, SCP2, ACAA2, ACOT2, ECI1, ECHS1, SCOT1, HADHA, HADHB and ACADVL)<br><br>Abundance of 93 mitochondrial proteins ↑ (ATPG, SCOT1, ALDH, THIM, NAD, NDUB9 etc.)<br><br>Proteins involved in cellular respiration, oxidative metabolism ↑<br><br>Proteins involved in TCA cycle, ETC activity ↑	Mitotracker™ intensity ↑ (1.5-fold)<br><br>Developed cristae ↑<br><br>Mitochondrial DNA ↑ (3-fold)<br><br>Mitochondrial proteome similarity to non-failing adult heart ↑		Production of ^{14}C-labeled CO_2 from glucose oxidation ↑ (+230 %)<br><br>Glucose consumption ↓ (2.5-fold)	Lactate production ↓ (4-fold)<br><br>Production of ^{14}C-labeled CO_2 from lactate oxidation ↑ (+388 %)<br><br>Glycogen deposits ↓	↑<br><br>Production of ^{14}C-labeled CO_2 from FAO ↑ (+214 %)	ATP synthesis ↑<br><br>ATP production from mitochondrial respiration ↑ (2.3-fold) (no statistics)<br><br>ATP production from anaerobic glycolysis ↓ (3.5-fold) (no statistics)<br><br>ATP production from glucose oxidation ↑ (1.6-fold) (no statistics)	

Table S2: Metabolic characteristics of HCM iPSC-CMs

Author	Culture method	Disease	Mutation	Control	Hypertrophic characteristics	Gene expression	Protein levels	Mitochondria	Respiratory capacity	Glucose metabolism	Fatty acid metabolism	ATP	Other cycles and pathways
Bhagwan et al. (2020)	2D (monolayer) + 3D (hEHTs)	HCM	*MYH7* (p.β-MHC-R453C(m)), α-actin (p.*ACTC1*-E99K(a))	Isogenic	Increase in cell volume ↑<br><br>Activation of BNP pathway<br><br>Sarcomeric disarray ↑<br><br>Translocation NFAT to nucleus ↑				Basal respiration ↑ (52% for *MYH7* heterozygous; 20% for *MYH7* homozygous; 2.6-fold for *ACTC1*)<br><br>Maximal respiration ↑ (1.35-fold for *MYH7* heterozygous, 1.43-fold for *MYH7* homozygous; 1.93-fold for *ACTC1*)				
Chou et al. (2017)	2D (monolayer)	Fabry disease	*GLA* (IVS4 + 919G > A)	One healthy control	Cell size ↑<br><br>Hypertrophy genes ↑	*CPT1A, CPT1B, CPT2, PDK4* ↓	AMPK phosphorylation, GLUT4 ↑		Maximal OCR ↓ (~2.1-fold)	Absolute ECAR ↓ (~2.3-fold)<br><br>Glycolysis ↓ (~3.3-fold)	FAO (Seahorse) ↓ (~3.3-fold)		GLA activity ↓ (~34.8-fold) (no statistics)

Table S2: Metabolic characteristics of HCM iPSC-CMs

Author	Culture method	Disease	Mutation	Control	Hypertrophic characteristics	Gene expression	Protein levels	Mitochondria	Respiratory capacity	Glucose metabolism	Fatty acid metabolism	ATP	Other cycles and pathways
Cohn et al. (2019)	3D (CMTs)	HCM	*MYBPC3* (Trp792ValfsX41; *MYBPC3*$^{+/-}$ & R502W), *MYH7* (R403Q & V606M)	Isogenic	Cell area ↑<br><br>Hypertrophy proteins ↑<br><br>Sarcomere disarray ↑	*MT-TP, MT-ND6, MT-ATP6P1, MT-ND4L, MT-ATP8, MT-RNR1, MT-ND2* ↑		Mitotracker™ intensity ↑ (~1.9-fold for R403Q, ~1.6-fold for *MYBPC3*$^{+/-}$, ~1.2-fold for R502W)				ADP/ATP ratio ↑ (~1.3-fold for R403Q)	ROS production ↑ (~3.3-fold for R403Q)
Hashem et al. (2017)	2D (monolayer) + long-term culture until day 60	Danon disease	*LAMP-2* (129-130 insAT (A), IVS-1 c.64 + 1G>A (B))	Two controls, pooled, identity not specified				Mitochondrial membrane potential ↓ (~1.9-fold)<br><br>Parkin staining ↑ (~5.3-fold)<br><br>p62 staining ↑ (~3.5-fold)<br><br>Abnormal mitochondria ↑ (~2.4-fold)	Maximal respiration ↓ (~1.7-fold for (A) and ~5.2-fold for (B))<br><br>ATP-linked respiration ↓ (~1.4-fold for (A) and ~4.7-fold for (B))				

Table S2: Metabolic characteristics of HCM iPSC-CMs

Author	Culture method	Disease	Mutation	Control	Hypertrophic characteristics	Gene expression	Protein levels	Mitochondria	Respiratory capacity	Glucose metabolism	Fatty acid metabolism	ATP	Other cycles and pathways
Hinson et al. (2016)	2D (monolayer) + 3D (CMTs)	PRKAG2 cardiac syndrome	*PRKAG2* (N488I, two patients: $P_A^{N488I/WT}$ (1) and $P_B^{N488I/WT}$(2))<br><br>Isogenic lines: $P_{AT}^{N488I/WT}$(3), $P_{AT}^{KO/KO}$ (4)<br><br>*All measurements concern (3) versus (7)*	Related control ($P_{c1}^{WT//WT}$ (5)), unrelated control ($P_{c2}^{WT//WT}$ (6)), isogenic control ($P_{AT}^{WT/WT}$ (7))	Cell size ↑<br><br>Hypertrophy genes ↑	Mitochondrial biogenesis and oxidative metabolism ↑ *PPARGCA1, PPARG, PPARA, HNF4A, ESRRA*<br><br>Glucose metabolism *SLC2A4, GYS1*↑, *SLC2A1* ↓ for<br><br>Fatty acid metabolism *CD36, FABP3*↑		Mitochondrial content ↑ (~1,1-fold)	Basal OCR ↑ (~1,5-fold)<br><br>Maximal OCR ↑ (~2,2-fold)	Glucose uptake ↑ (8,3%)<br><br>Lactate production ↓ (8,3%)<br><br>Intracellular glycogen ↑ (~1,2-fold for (3)), ↓ (~1,1-fold for (4))<br><br>Glycolysis intermediates G6P ↑, F6P, 1,3-BPG, 3PG ↓<br><br>Glycogen precursor G1P ↑<br><br>Isoform switch in PFK-1 to less active form<br><br>Isoform switch in GP to less active form	Carnitine, C2-, C5-, C8-long chain acylcarnitines, long chain acyl-CoA ↑		

Table S2: Metabolic characteristics of HCM iPSC-CMs

Author	Culture method	Disease	Mutation	Control	Hypertrophic characteristics	Gene expression	Protein levels	Mitochondria	Respiratory capacity	Glucose metabolism	Fatty acid metabolism	ATP	Other cycles and pathways
Kargaran et al. (2020)	2D (monolayer)	HCM	*MYH7* (R453C)<br><br>Heterozygous (mut/wt), homozygous (mut/mut), knock-out (ko/ko)<br><br>In 2 lines (AT1 and REBL-PAT)	Isogenic	(see Mosquiera et al. (2018))			AT1:<br>*MT-ND1* ↓ (1.2-fold for *MYH7*$^{mut/wt}$ (n.s), 1.2-fold for *MYH7* $^{mut/mut}$ (n.s), 1.6-fold for *MYH7*$^{ko/ko}$)<br><br>*MT-ND2* ↓ (1.1 for *MYH7*$^{mut/wt}$ (n.s.), ± for *MYH7* $^{mut/mut}$, 1.9-fold for *MYH7*$^{ko/ko}$)<br><br>REBL-PAT:<br>*MT-ND1* ↓ (1.1-fold for *MYH7*$^{mut/wt}$ (n.s), 1.3-fold for *MYH7* $^{mut/mut}$ (n.s))<br><br>*MT-ND2* ↓ (1.3-fold for *MYH7*$^{mut/wt}$ (n.s), 1.6-fold for *MYH7* $^{mut/mut}$)					

Table S2: Metabolic characteristics of HCM iPSC-CMs

Author	Culture method	Disease	Mutation	Control	Hypertrophic characteristics	Gene expression	Protein levels	Mitochondria	Respiratory capacity	Glucose metabolism	Fatty acid metabolism	ATP	Other cycles and pathways
Kargaran et al. (2020) *cont.*		HCM	*ACTC1* (p.E99K)	Isogenic, gene corrected (GC) + mutation introduced in healthy relative (NC) *Depicted values are (NC) compared to (GC)*					Basal respiration ↑ (1.6-fold)<br><br>Maximal respiration ↑ (1.7-fold)<br><br>Spare capacity ↑ (2-fold)<br><br>Proton leak ↑ (1.1-fold) (n.s.)			ATP production ↑ (1.7-fold)	

Table S2: Metabolic characteristics of HCM iPSC-CMs

Author	Culture method	Disease	Mutation	Control	Hypertrophic characteristics	Gene expression	Protein levels	Mitochondria	Respiratory capacity	Glucose metabolism	Fatty acid metabolism	ATP	Other cycles and pathways
Li et al. (2018)	2D (monolayer) + long-term culture until day 40	Mitochondrial cardiomyopathy	*MT-RNR2* (m.2336T>C)	Pro-band's son and two healthy unrelated controls from the same region	Cell size ↑<br><br>Hypertrophy proteins ↑	*16s rRNA* ↓ *p.MT-ND5, p.MT-CYB, p.MT-CO2, p.MT-ATP8* ↓	MRPL19, MRPL23 ↓ (51.1% and 23.8%)	Mitochondria round and immature, underdeveloped cristae<br><br>Few elongated mitochondria with developed cristae<br><br>Mitochondrial content (TEM) ↑ (1.3-fold)<br><br>Mitochondrial potential ↓ (25%)<br><br>mtDNA copy number ↑ (71%)				ATP/ADP ratio ↓ (47%)	

Table S2: Metabolic characteristics of HCM iPSC-CMs

Author	Culture method	Disease	Mutation	Control	Hypertrophic characteristics	Gene expression	Protein levels	Mitochondria	Respiratory capacity	Glucose metabolism	Fatty acid metabolism	ATP	Other cycles and pathways
Mosqueira et al. (2018)	2D (monolayer) + 3D (EHTs)	HCM	*MYH7* (c.C9123T-MYH7, R453C) in 3 lines (AT1, REBL-PAT, HUES7)<br><br>*All values depicted are in AT-1 lines*<br><br>C9123T mutation types: heterozygous ((1)), homozygous ((2)), homozygous with frameshift in *MYH6* ((3)), knockout ((4))<br><br>*All conditions are compared to wild-type*	Isogenic	Cell size ↑<br><br>Hypertrophy proteins ↑			*MT-ND1* ↓ (~1.2-fold for (1) (n.s.), ± for (2), ~1.4-fold for (3) (n.s.), ~1.6-fold for (4))<br><br>*MT-ND2* ↓ (~1.2-fold for (1) (n.s.), ~1.2-fold for (2) (n.s.), ~1.4-fold for (3) (n.s.), ~2-fold for (4))<br><br>Mitotracker™ ↓ (± for (1), ± for (2), 1.1-fold for (3) (n.s.), 1.2-fold for (4) (n.s.))	Basal respiration ↑ (1.9-fold for (1), 2-fold for (2), 2.3-fold for (3), 2.3-fold for (4))<br><br>Maximal respiration ↑ (1.7-fold for (1), 2.2-fold for (2), 2.6-fold for (3), 2.2-fold for (4))			ATP produc-tion ↑ (2.4-fold for (1), 2.8-fold for (2), 2.3-fold for (3), 3-fold for (4))	ROS production:<br><br>CellROX™ ↑ (1.1-fold for (1) (n.s.), 1.5-fold for (2), ± for (3), 1.5-fold for (4) (n.s.))<br><br>MitoSOX™ ↑ (1.1 for (1), 1.2-fold for (2) (n.s.), 1.3-fold for (3) (n.s.), 1.5-fold for (4) (n.s.))

Table S2: Metabolic characteristics of HCM iPSC-CMs

Author	Culture method	Disease	Mutation	Control	Hypertrophic characteristics	Gene expression	Protein levels	Mitochondria	Respiratory capacity	Glucose metabolism	Fatty acid metabolism	ATP	Other cycles and pathways
Pua et al. (2020)	2D (monolayer)	HCM	*TNNT2* (R286H), *MYH7* (R403Q)		Cell size ↑				OCR ↑ (1.2-fold for *TNNT2*, 3.1-fold for *MYH7*)	ECAR ↑ (1.3-fold for *TNNT2*, 1.6-fold for *MYH7*)			
Ramachandra et al. (2021)	2D (cardiac clusters)	HCM	*MYBPC3* (D389V), *MYH7* (R243C)	Healthy subjects	Cell size ↑<br><br>Hypertrophy proteins ↑<br><br>Translocation NFAT to nucleus ↑	*MPO* transcripts ↑	MPO protein levels ↑						Chlorination ↑(2.3-fold for *MYBPC3*, 2.4-fold for *MYH7*)<br><br>Peroxidation ↑ (2-fold for *MYBPC3*, 2.3-fold for *MYH7*)<br><br>ROS levels ↑

Table S2: Metabolic characteristics of HCM iPSC-CMs

Author	Culture method	Disease	Mutation	Control	Hypertrophic characteristics	Gene expression	Protein levels	Mitochondria	Respiratory capacity	Glucose metabolism	Fatty acid metabolism	ATP	Other cycles and pathways
Toepfer et al. (2020)	2D (monolayer)	HCM	*MYH7* (R403Q, V606M, R719W)	Isogenic	Cell size ↑<br><br>Sarcomere content ↑			Mitotracker™ intensity ↑ (1.4-fold for R403Q, 1.7-fold for V606M, 1.7-fold for R719W)	Capacity for maximal respiration ↑<br><br>Basal OCR ↑ (3.1-fold for R403Q, 2.4-fold for V606M and R719W)<br><br>Basal OCR when cultured in glucose ↓ (1.1-fold for all variants) (n.s.)	Glucose, glucose-6-P, Fructose-1,6-P, 3-Phosphoglycerate, PEP, lactate ↑ (R403Q)		ATP content ±<br><br>PCr/ATP ratio ↓ (2.3-fold for *MYH7* variants com-bined)	Succinic acid, fumaric acid, aconitic acid, citric acid ↑ (*MYH7*)<br><br>NAD^+/NADH ratio ↑ (2.1-fold for *MYH7* variants combined)

Table S2: Metabolic characteristics of HCM iPSC-CMs

Author	Culture method	Disease	Mutation	Control	Hypertrophic characteristics	Gene expression	Protein levels	Mitochondria	Respiratory capacity	Glucose metabolism	Fatty acid metabolism	ATP	Other cycles and pathways
Wang et al. (2014)	2D (monolayer)	Barth syndrome	*TAZ* (c.517delG (BTH-H), c.238T>C (BTH-C))	Three healthy iPSC lines				Mitochondrial potential ↑ (1,5-fold)	For BTH-H:<br>Basal OCR ↑ (2.2-fold)<br><br>Reserve respiration capacity ↓ (3.5-fold)<br><br>Proton leak ↑ (2-fold)<br><br>ATP synthase OCR ↑ (4.9-fold)	For BTH-H:<br>OCR/ECAR ratio (in galactose) ↑ (1.5-fold)<br><br>OCR/ECAR ratio (in glucose) ↓ (2.3-fold)		ATP content ↓ (2.6-fold for BTH-H, 2.4-fold for BTH-C)	Mitochondrial ROS ↑ (4.7-fold for BTH-H)
Zhan et al. (2018)	2D (cardiac clusters)	PRKAG2 cardiac syndrome	*PRKAG2* (R302Q)<br>2 patients: ZW & ZJ (w/o hypertrophy)	Healthy unrelated control, gender- and age-matched	Cell size ↑<br><br>Sarcomere disarray ↑	*NRF1*, *PPARA* and *TFAM* ↑<br>*CD36* ↑<br>*PPARCG1A* ↑ (ZW)<br>*PPARG* ↑			Basal respiration ↑ (~1.1-fold for both ZW and ZJ) (n.s.)<br><br>Maximal respiration ↑ (~1.5-fold for ZW and ~1.2-fold for ZJ)	Glycogen synthase activity ↑ (~1.6-fold for ZW)<br><br>Glycogen content ↑ (~1.9-fold for ZW and ~1.4-fold for ZJ)			AMPK activity ↑ (~1.7-fold for ZW, ~1.9-fold for ZJ)

Table S2: Metabolic characteristics of HCM iPSC-CMs

Author	Culture method	Disease	Mutation	Control	Hypertrophic characteristics	Gene expression	Protein levels	Mitochondria	Respiratory capacity	Glucose metabolism	Fatty acid metabolism	ATP	Other cycles and pathways
Zhan et al. (2018) *cont.*									Respiration capacity ↑ (~2.1-fold for ZW and ~1.6-fold for ZJ)<br><br>ATP-linked respiration ↑ (~1.4-fold for ZW, ~1,2-fold for ZJ (n.s.))				
